# Supplementary material for: Factors associated with soil-transmitted helminths infection in Benin: Findings from the DeWorm3 study
Source: PLoS Negl Trop Dis. 2021 Aug 17;15(8):e0009646. doi: 10.1371/journal.pntd.0009646 (PMC8396766; doi:10.1371/journal.pntd.0009646)
Supplement: S1 Table — (DOCX) [file pntd.0009646.s002.docx]

| **SUPPLEMENTARY DATA** |
| --- |

**S1 Table: Summary of Kato-Katz Quality Assurance (QA)**

| Selected for QA: n | 1,270 |
| --- | --- |
| Completion of Kato-Katz QA: n (%) | |
| Sample selected for QA and tested | 1,227 (95.6) |
| Sample selected for QA but was not tested | 43 (3.4) |
| Sample was not selected for QA but was tested | 13 (1.0) |
| Agreement between original Kato-Katz reading and QA reading: n (%) | |
| Original and QA readings agree | 1,219 (99.3) |
| Original and QA readings do not agree^1^ | 8 (0.7) |

Notes: ^1^ n=4 slides were hookworm positive in the original reading and hookworm negative in the QA reading, n=2 were *Ascaris lumbricoides* positive in the original reading and *Ascaris lumbricoides* negative in the QA reading, n=1 were *Ascaris* negative in the original reading and *Ascaris lumbricoides* positive in the QA reading, and n=1 were *Trichuris trichiura* negative in the original reading and *Trichuris trichiura* positive in the QA reading.
