# Supplementary material for: Factors associated with soil-transmitted helminths infection in Benin: Findings from the DeWorm3 study
Source: PLoS Negl Trop Dis. 2021 Aug 17;15(8):e0009646. doi: 10.1371/journal.pntd.0009646 (PMC8396766; doi:10.1371/journal.pntd.0009646)
Supplement: S2 Table — (DOCX) [file pntd.0009646.s003.docx]

| **SUPPLEMENTARY DATA** |
| --- |

**S2 Table: Burden of moderate-to-heavy intensity (MHI) STH infection in the study population by age group, during DeWorm3 baseline analysis in Comé, Bénin**

| Age group | n = 6,139 | MHI  Any STH | MHI Hookworm | MHI  *Ascaris lumbricoides* | MHI *Trichuris trichiura* |
| --- | --- | --- | --- | --- | --- |
| Adults (≥15 y ) | 3,620 | 24 (0.7%) | 7 (0.2) | 17 (0.5%) | 0 |
| PSAC (1-4 y) | 1,335 | 14 (1.1%) | 1 (0.1%) | 13 (1.0%) | 0 |
| SAC (5-14 y) | 1,184 | 28 (2.3%) | 2 (0.2%) | 25 (2.1%) | 1 (0.0) |
