# Supplementary material for: Factors associated with soil-transmitted helminths infection in Benin: Findings from the DeWorm3 study
Source: PLoS Negl Trop Dis. 2021 Aug 17;15(8):e0009646. doi: 10.1371/journal.pntd.0009646 (PMC8396766; doi:10.1371/journal.pntd.0009646)
Supplement: S3 Table — (DOCX) [file pntd.0009646.s004.docx]

| **SUPPLEMENTARY DATA** |
| --- |

**S3 Table: Burden of moderate-to-high intensity STH infection among infected individuals by age group and gender, during DeWorm3 baseline analysis in Comé, Bénin**

|  | n=66 | Hookworm | *Ascaris lumbricoides* | *Trichuris trichiura* | Total (%) |
| --- | --- | --- | --- | --- | --- |
| PSAC | Male | 1 | 5 | - | 6 (9) |
|  | Female | - | 8 | - | 8 (12.1) |
| SAC | Male | 2 | 20 | 1 | 23 (34.8) |
|  | Female | - | 5 | - | 5 (7.5) |
| Adults | Male | 4 | 12 | - | 16 (24.2) |
|  | Female | 3 | 5 | - | 8 (12.1) |
| Total (%) |  | 10 (15.1) | 55 (83.3) | 1(1.5) | 66 (100) |
