# Supplementary material for: Factors associated with soil-transmitted helminths infection in Benin: Findings from the DeWorm3 study
Source: PLoS Negl Trop Dis. 2021 Aug 17;15(8):e0009646. doi: 10.1371/journal.pntd.0009646 (PMC8396766; doi:10.1371/journal.pntd.0009646)
Supplement: S4 Table — (DOCX) [file pntd.0009646.s005.docx]

| **SUPPLEMENTARY DATA** |
| --- |

**S4 Table: Factors univariately associated with hookworm infection in Comé, Bénin: findings from DeWorm3 cluster randomized trial baseline pre-treatment survey using generalized logistic mixed model.**

|  |  |  | Factors associated with prevalence of hookworm infection | | Factors associated with intensity of hookworm infection | |
| --- | --- | --- | --- | --- | --- | --- |
| Variables | Infected by hookworm | Intensity of hookworm infection | Univariate Logistic regression Analysis |  | Univariate negative binomial analysis |  |
|  | n (%) | Median (IQR), [min, max] epg | OR (95% CI) | p-value | IIR (95% CI) | p-value |
| Individual factors |  |  |  |  |  |  |
| Age | n = 6,139 | n = 6,138 |  | <0.001 |  | <0.0001 |
| - Adults (≥15 years ) | 160 (4.4) | 0(0-0), [0-12,960] | Reference |  | Reference |  |
| - PSAC (1-4 years) | 27 (2.0) | 0 (0-0), [0-3,552] | 0.1 (0.1-0.3) | <0.001 | 0.04 (0.01 -0.13) | <0.001 |
| - SAC (5-14 years) | 12 (1.0) | 0 (0-0), [0-11,100] | 0.3 (0.2-0.6) | <0.001 | 0.1 (0.0-0.3) | <0.001 |
| Gender | n = 6,139 | n = 6,138 |  | 0.002 |  | 0.0003 |
| - Male | 113 (4.0) | 0 (0-0), [0-12,960] | Reference |  | Reference |  |
| - Female | 86 (2.6) | 0 (0-0), [0-9,840] | 0.6 (0.4-0.8) | 0.002 | 0.3 (0.1-0.6) | <0.001 |
| History of deworming during the past year | n = 6,091 | n = 6,090 |  | <0.001 |  | <0.0001 |
| - No | 171 (4.6) | 0 (0-0), [0-12,960] | Reference |  | Reference |  |
| - Yes | 28 (1.2) | 0 (0-0), [0-3,048] | 0.2 (0.1-0.4) | <0.001 | 0.05 (0.02-0.12) | <0.001 |
| Shoe wearing behavior | n = 6,091 | n = 6,090 |  | 0.85 |  | 0.96 |
| - Shoes | 100 (3.0) | 0 (0-0), [0-12,960] | Reference |  | Reference |  |
| - No shoes | 99 (3.6) | 0 (0-0), [0-11,100] | 1.0 (0.7-1.5) | 0.85 | 1.0 (0.5-1.9) | 0.96 |
| Current school attendance | n = 6,139 | n=6,138 |  | 0.29 |  | 0.18 |
| - No | 152 (3.5) | 0(0-0), [0-12,960] | Reference |  | Reference |  |
| - Yes | 47 (2.5) | 0(0-0), [0-11,100] | 0.8 (0.5-1.2) | 0.29 | 0.6 (0.3-1.3) | 0.18 |
|  |  |  |  |  |  |  |
| Household factors |  |  |  |  |  |  |
| Highest education level in the household | n = 6,139 | n = 6,138 |  | 0.002 |  | 0.0015 |
| - University/College/Diploma | 4 (0.6) | 0(0-0), [0-228] | Reference |  | Reference |  |
| - No education | 93 (4.8) | 0(0-0), [0-12,960] | 6.8 (2.2-20.9) | 0.001 | 39.7 (5.6-279.2) | <0.001 |
| - Primary | 50 (3.7) | 0(0-0), [0-11,100] | 5.0 (1.6-15.6) | 0.01 | 26.6 (3.7-191.6) | 0.001 |
| - Secondary | 52 (2.4) | 0(0-0), [0-4,764] | 3.7 (1.2-11.2) | 0.02 | 15.9 (2.3-109.0) | 0.005 |
| Quintiles of household asset index | n = 6,139 | n = 6,138 |  | <0.001 |  | <0.0001 |
| - 5th quintile (highest) | 16 (1.0) | 0(0-0), [0-1,440] | Reference |  | Reference |  |
| - 1st quintile (lowest) | 76 (7.7) | 0(0-0), [0-12,960] | 7.1 (3.4-14.8) | <0.001 | 53.5 (15.2-188.8) | <0.001 |
| - 2nd quintile | 51 (4.9) | 0(0-0), [0-4,764] | 4.8 (2.4-9.9) | <0.001 | 22.0 (6.3-76.4) | <0.001 |
| - 3rd quintile | 38 (3.2) | 0(0-0) [0-9,840] | 2.8 (1.4-5.7) | 0.01 | 7.1 (2.1-24.1) | 0.002 |
| - 4th quintile | 18 (1.3) | 0(0-0), [0-1,104] | 1.2 (0.5-2.5) | 0.66 | 1.3 (0.4-4.8) | 0.67 |
| Head of household’s occupation | n = 6,139 | n = 6,138 |  | <0.0001 |  | <0.0001 |
| - Others/ Don’t know/Refused | 98 (2.1) | 0(0-0), [0-4,764] | Reference |  | Reference |  |
| - Farmer | 89 (9.7) | 0(0-0), [0-12,960] | 3.6 (2.2-6.0) | <0.001 | 18.1 (7.4-44.3) | <0.001 |
| - Fisher | 12 (2.0) | 0(0-0), [0-516] | 1.2 (0.5-2.5) | 0.69 | 0.7 (0.2-3.1) | 0.67 |
| Observed floor type : natural/manmade | n = 6,139 | n = 6,138 |  | <0.0001 |  | <0.0001 |
| - Man-made floor material | 123 (2.4) | 0(0-0), [0-11,100] | Reference |  | Reference |  |
| - Natural floor material | 75 (7.9) | 0(0-0),[0-12,960] | 2.9 (1.9-4.5) | <0.001 | 11.5 (5.1-25.7) | <0.001 |
| - Other/Don’t know/Refused | 1 (3.7) | 0(0-0), [0-48] | 2.9 (0.2-35.6) | 0.40 | 4.9 (0.0-1068.6) | 0.56 |
| Urbanization | n = 6,134 | n = 6,133 |  | <0.0001 |  | <0.0001 |
| - Urban | 29 (1.2) | 0(0-0), [0-9,840] | Reference |  | Reference |  |
| - Peri-urban | 146 (5.0) | 0(0-0), [0-12,960] | 4.3 (2.3-8.2) | <0.0001 | 22.5 (7.1-70.6) | <0.001 |
| - Rural | 24 (3.0) | 0(0-0), [0-3,120] | 2.7 (1.1-6.6) | 0.03 | 6.3 (1.2-32.7) | 0.03 |
| Population density at 1km | n = 6,134 | n = 6,133 |  | <0.0001 |  | <0.0001 |
| 1^st^ tertile [3 ; 542[ (lowest) | 135 (6.7) | 0(0-0) [0-12,960] | Reference |  | Reference |  |
| 2^nd^ tertile [542 ; 1235[ | 43 (2.1) | 0(0-0), [0-3,624] | 0.4 (0.2-0.7) | 0.001 | 0.1 (0.0-0.4) | <0.001 |
| 3^rd^ tertile [1235 ; 2528] (highest) | 21 (1.0) | 0(0-0), [0-9,840] | 0.1 (0.7-0.3) | < 0.001 | 0.02 (0.0-0.1) | <0.001 |
|  |  |  |  |  |  |  |
| WASH factors |  |  |  |  |  |  |
| Household water service | n = 6,135 | n = 6,134 |  | 0.001 |  | - |
| - Improved ≤ 30min | 141 (2.8) | 0 (0-0),[0-2,124] | Reference |  | Reference |  |
| - Surface water > 30min | 0 (0.0) | 0(0-0), [0-3120] | 0 | - | 0 | - |
| - Surface water ≤ 30min | 2 (12.5) | 0(0-0), [0-120] | 11.1 (1.2-103.7) | 0.03 | 75.7 (1.3-4390.3) | 0.04 |
| - Unimproved > 30min | 6 (11.3) | 0(0-0), [0-696] | 5.8 (1.6-21.1) | 0.01 | 97.7 (8.8-1085.2) | <0.001 |
| - Unimproved ≤ 30min | 39 (6.7) | 0(0-0), [0-2,124] | 2.7 (1.5-4.8) | 0.001 | 10.7 (3.9-29.3) | <0.001 |
| - Improved > 30 min | 11 (2.9) | 0(0-0), [0-3,120] | 1.0 (0.4-2.1) | 0.91 | 0.9 (0.2-4.1) | 0.95 |
| Household sanitation service | n = 5,816 | n = 5,815 |  | 0.0001 |  | <0.0001 |
| - Open defecation | 129 (6.0) | 0(0-0), [0-12,960] | Reference | 0.04 | Reference |  |
| - Unimproved shared | 3 (1.3) | 0(0-0), [0-60] | 0.2 (0.1-1.0) | 0.06 | 0.03 (0.002-0.39) | 0.01 |
| - Unimproved unshared | 1 (0.8) | 0(0-0), [0-204] | 0.1 (0.0-1.1) | <0.001 | 0.02 (0.0006-0.54) | 0.02 |
| - Improved shared | 28 (1.7) | 0(0-0), [50-9,840] | 0.3 (0.2-0.5) | <0.001 | 0.1 (0.0-0.2) | <0.001 |
| - Improved unshared | 28 (1.7) | 0(0-0), [0-1,440] | 0.3 (0.1-0.5) | 0.17 | 0.1 (0.0-0.2) | <0.001 |
| Household hand washing facility service | n = 5,716 | n = 5,715 |  |  |  | 0.007 |
| - No facility | 64 (3.4) | 0(0-0), [0-12,960] | Reference |  | Reference |  |
| - Limited | 104 (3.3) | 0(0-0), [0-8,064] | 1.2 (0.8-1.8) | 0.45 | 1.3 (0.6-2.9) | 0.51 |
| - Basic | 13 (1.8) | 0(0-0), [0-1,440] | 0.6 (0.3-1.2) | 0.17 | 0.3 (0.1-1.2) | 0.08 |
|  |  |  |  |  |  |  |
| Environmental Factors |  |  |  |  |  |  |
| Elevation (in meters) | n = 6,134 | n = 6,133 |  | 0.02 |  | 0.003 |
| 1^st^ tertile [-1 ; 15[ | 63 (3.0) | 0(0-0), [0-12,960] | Reference |  | Reference |  |
| 2^nd^ tertile [15 ; 30[ | 37 (1.6) | 0(0-0), [0-9,840] | 0.4 (0.2-0.9) | 0.02 | 0.2 (0.0-0.5) | 0.003 |
| 3^rd^ tertile [30 ; 61] | 99 (5.7) | 0(0-0), [0-11,100] | 1.0 (0.5-1.8) | 0.97 | 0.9 (0.3-2.6) | 0.79 |
| Soil sand fraction at the surface at 0 cm (%) | n = 6,134 | n = 6,133 |  | 0.01 |  | 0.001 |
| 1^st^ tertile [35 ; 55[ | 37 (1.7) | 0(0-0), [0-1,176] | Reference |  | Reference |  |
| 2^nd^ tertile [55 ; 64[ | 40 (2.0) | 0(0-0), [0-3,624] | 1.1 (0.6-2.1) | 0.67 | 1.5 (0.5-4.6) | 0.48 |
| 3^rd^ tertile [64 ; 78] | 122 (6.1) | 0(0-0), [0-12,960] | 2.4 (1.3-4.7) | 0.006 | 8.6 (2.5-29.2) | 0.001 |
| Soil acidity at everage depth (0-5-15 cm) | n = 6,134 | n = 6,133 |  | 0.0351 |  | 0.02 |
| 1^st^ tertile [4.8 ; 5.1[ | 40 (2.0) | 0(0-0), [0-11,100] | Reference |  | Reference |  |
| 2^nd^ tertile [5.1 ; 5.2[ | 90 (4.0) | 0(0-0), [0-9,840] | 1.9 (1.1-3.2) | 0.01 | 3.5 (1.3-9.2) | 0.01 |
| 3^rd^ tertile [5.2 ; 5.7] | 69 (3.7) | 0(0-0), [0-12,960] | 1.9 (1.1-3.2) | 0.03 | 4.0 (1.4-11.5) | 0.01 |
| MODIS daytime land surface temperature mean for 2018 (°celsius) | n = 6,134 | n = 6,133 |  | 0.0082 |  | 0.0002 |
| 1^st^ tertile [26.2 ; 29.6[ | 71 (3.4) | 0(0-0), [0-11,100] | Reference |  | Reference |  |
| 2^nd^ tertile [29.6 ; 31.9[ | 109 (4.5) | 0(0-0), [0-12,960] | 1.2 (0.7-2.2) | 0.43 | 2.7 (1.0-7.4) | 0.06 |
| 3^rd^ tertile [31.9 ; 32.8] | 19 (1.2) | 0(0-0), [0-3,624] | 0.3 (0.1-0.8) | 0.02 | 0.1 (0.02-0.6) | 0.01 |
| MODIS Enhanced Vegetation Index (EVI) mean for 2018 | n =6,134 | n = 6,133 |  | < 0.0001 |  | <0.0001 |
| 1^st^ tertile [0.04 ; 0.2[ | 26 (1.2) | (0-0), [0-9,840] | Reference |  | Reference |  |
| 2^nd^ tertile [0.2 ; 0.3[ | 35 (1.7) | (0-0), [0-3,624] | 1.4 (0.7-2.7) | 0.29 | 2.2 (0.7-7.1) | 0.17 |
| 3^rd^ tertile [0.3 ; 0.4] | 138 (6.9) | (0-0), [0-12,960] | 5.2 (2.6-10.4) | < 0.001 | 42.4 (11.9-151.4) | <0.001 |
| MODIS normalized difference vegetation index (NDVI) mean for 2018 | n = 6,134 | n = 6,133 |  | < 0.0001 |  | <0.0001 |
| 1^st^ tertile [0.06 ; 0.3[ | 27 (1.3) | (0-0), [0-9,840] | Reference |  | Reference |  |
| 2^nd^ tertile [0.3 ; 0.4[ | 33 (1.6) | (0-0), [0-3,624] | 1.2 (0.6-2.3) | 0.57 | 1.6 (0.5-4.9) | 0.44 |
| 3^rd^ tertile [0.4 ; 0.6] | 139 (7.0) | (0-0), [0-12,960] | 4.9 (2.5-9.8) | < 0.001 | 36.2 (10.2-127.7) | <0.001 |
| Aridity index | n = 6,134 | n = 6,133 |  | 0.28 |  | 0.10 |
| 1^st^ tertile [0.59 ; 0.61[ | 81 (3.9) | (0-0), [0-9,840] | Reference |  | Reference |  |
| 2^nd^ tertile [0.61 ; 0.62[ | 50 (2.4) | (0-0), [0-8,064] | 1.2 (0.6-2.4) | 0.57 | 1.1 (0.3-4.2) | 0.87 |
| 3^rd^ tertile [0.65 ; 0.65] | 68 (3.5) | (0-0), [0-12,960] | 1.8 (0.8-4.0) | 0.12 | 3.6 (0.9-14.8) | 0.07 |

Notes:

†Generalized estimating equations with exchangeable correlation structure and logit link applied

All data available displayed for “number infected with Hookworm (prevalence [%])”

Acronyms: School Aged Children (SAC), Pre School Aged Children (PSAC), confidence interval (CI), odds ratio (OR), Infection Intensity Ratio (IIR), Moderate Resolution Imaging Spectroradiometer (MODIS)
