# Supplementary material for: Factors associated with soil-transmitted helminths infection in Benin: Findings from the DeWorm3 study
Source: PLoS Negl Trop Dis. 2021 Aug 17;15(8):e0009646. doi: 10.1371/journal.pntd.0009646 (PMC8396766; doi:10.1371/journal.pntd.0009646)
Supplement: S5 Table — (DOCX) [file pntd.0009646.s006.docx]

| **SUPPLEMENTARY DATA** |
| --- |

**S5 Table: Intra-Class Correlation values**

| Risk factor analysis | Model | Level of clustering | Intra-Class Correlation (95% IC)* |
| --- | --- | --- | --- |
| Hookworm infection prevalence** | Model with age and sex | Cluster level | 0.16 (0.10-0.26) |
|  |  | Household within Cluster level | 0.58 (0.40-0.74) |
|  | Fully adjusted model | Cluster level | 0.03 (0.01-0.10) |
|  |  | Household within Cluster level | 0.39 (0.18-0.65) |
| Ascaris lumbricoides infection prevalence ** | Model with age and sex | Cluster level | 0.54 (0.34-0.73) |
|  |  | Household within Cluster level | 0.60 (0.38-0.78) |
|  | Fully adjusted model | Cluster level | 0.42 (0.23-0.64) |
|  |  | Household within Cluster level | 0.51 (0.26-0.76) |

Notes:

*The Intraclass correlation statistics reports two intraclass correlations for this three-level nested model. The first is the level-3 intraclass correlation at the cluster level, the correlation between Hookworm or *Ascaris lumbricoides* infection prevalence in the same cluster. The second is the level-2 intraclass correlation at the household-within-cluster level, the correlation between prevalence of infection with *Ascaris lumbricoides* in the same household and cluster.

** Generalized logistic mixed model with exchangeable correlation matrix
