# Supplementary material for: Factors associated with soil-transmitted helminths infection in Benin: Findings from the DeWorm3 study
Source: PLoS Negl Trop Dis. 2021 Aug 17;15(8):e0009646. doi: 10.1371/journal.pntd.0009646 (PMC8396766; doi:10.1371/journal.pntd.0009646)
Supplement: Attachment — Submitted filename: Avokpaho et al 2020_PNTD-D-20-02235.pdf [file pntd.0009646.s007.pdf]

PLOS Neglected Tropical Diseases

Factors associated with soil-transmitted helminth infection in Benin: findings from the DeWorm3 study

--Manuscript Draft--

**Manuscript Number:**	PNTD-D-20-02235
**Full Title:**	Factors associated with soil-transmitted helminth infection in Benin: findings from the DeWorm3 study
**Short Title:**	Deworm3 study findings of factors associated with STH infection in Benin.
**Article Type:**	Research Article
**Keywords:**	Soil Transmitted Helminths, prevalence, risk factor, DeWorm3, Comé, Benin
**Abstract:**	Background: Despite several years of school-based MDA implementation, STH remain an important public health problem in Benin, with a country-wide prevalence of 20% in 2015. The DeWorm3 study is designed to assess the feasibility of using community-based MDA with albendazole to interrupt the transmission of STH, through a series of cluster-randomized trials in Benin, India and Malawi. We used the pre-treatment baseline survey data to describe and analyze the factors associated with STH infection in Comé, the study site of the DeWorm3 project in Benin. These data will improve understanding of the challenges to be addressed in order to eliminate STH as a public health problem in Benin.<br>.<br>Methods: Between March and April 2018, the prevalence of STH (hookworm spp., Ascaris and Trichuris trichiura) was assessed by Kato-Katz in stool samples collected from 6,153 residents in the community of Comé, Benin using a stratified random sampling procedure . A standardized survey questionnaire was used to collect information from individual households concerning factors potentially associated with the presence and intensity of STH infections in pre-school (PSAC, aged 1-4), school-aged children (SAC, aged 5-14) and adults (aged 15 and above). Multilevel mixed-effects models were used to assess associations between these factors and STH infection.<br><br>Results: The overall prevalence of STH infection was 5.3%; 3.2% hookworm spp., 2.1% Ascaris and 0.1% Trichuris . Hookworm spp. were more prevalent in adults than in SAC (4.4% versus 2.0%, respectively; p=0.0001) and PSAC (4.4% versus 1.0%, respectively; p<0.0001), whilst Ascaris was more prevalent in SAC than in adults (3.0% versus 1.7%, respectively; p= 0.004). Being PSAC (adjusted Odds Ratio (aOR)=0.21, p< 0.001; adjusted Infection Intensity Ratio (aIIR) =0.10, p<0.001) or SAC (aOR=0.49, p=0.008; aIIR=0.29, p=0.01), being a female (aOR=0.56, p=0.004; aIIR=0.32, p=0.001), and having received deworming treatment the previous year (aOR= 0.45, p< 0.002; aIIR=0.20, p<0.001) were associated with a lower prevalence and intensity of hookworm infection. Lower income (lowest quintile: aOR= 5.03, p<0.001, 2 nd quintile aOR= 3.62, p=0.001 and 3 rd quintile aOR= 2.51, p=0.02), being a farmer (aOR= 1.79, p=0.02), medium population density (aOR= 2.59, p=0.01), and open defecation (aOR=0.48, p=0.04) were associated with a higher prevalence of hookworm infection. Lower education - no education, primary or secondary school- (aIIR=40.13, p=0.01; aIIR=30.92, p=0.02; aIIR=19.34, p=0.04, respectively), farming (aIIR=3.94, p=0.002), natural flooring (aIIR=0.23, p=0.06) , peri-urban settings (aIIR=6.18, 95%CI 1.82 - 20.90, p=0.003) , and unimproved water source more than 30 minutes from the household (aIIR= 13.47, p=0.02) were associated with a higher intensity of hookworm infection. Improved and unshared toilet was associated with lower intensity of hookworm infections (aIIR=0.23, p=0.01). SAC had a higher odds of Ascaris infection than adults (aOR= 2.0, p=0.01) and females had a lower odds of infection (aOR= 0.5, p= 0.02).<br><br>Conclusion:<br>Hookworm spp. are the most prevalent STH in Comé, with a persistent reservoir in adults that is not addressed by current control measures based on school MDA. Expanding MDA to target adults and PSAC is necessary to substantially impact population prevalence, particularly for hookworm.

Powered by Editorial Manager® and ProduXion Manager® from Aries Systems Corporation

Additional Information:	
**Question**	**Response**
**Financial Disclosure**<br><br>Enter a financial disclosure statement that describes the sources of funding for the work included in this submission. Review the submission guidelines for detailed requirements. View published research articles from *PLOS NTDs* for specific examples.<br><br>This statement is required for submission and **will appear in the published article** if the submission is accepted. Please make sure it is accurate.<br><br>**Unfunded studies**<br><br>Enter: *The author(s) received no specific funding for this work.*<br><br>**Funded studies**<br><br>Enter a statement with the following details:<br>• Initials of the authors who received each award<br>• Grant numbers awarded to each author<br>• The full name of each funder<br>• URL of each funder website<br>• Did the sponsors or funders play any role in the study design, data collection and analysis, decision to publish, or preparation of the manuscript?<br>• **NO** - Include this sentence at the end of your statement: *The funders had no role in study design, data collection and analysis, decision to publish, or preparation of the manuscript.*<br>• **YES** - Specify the role(s) played.<br><br>* typeset	JLW and TL received the DeWorm3 study funding from The Bill and Melinda Gates foundation (OPP1129535). https://www.gatesfoundation.org/. The funders had no role in study design, data collection and analysis, decision to publish, or preparation of the manuscript.<br><br>EA is a PhD candidate at the University of Paris. His research is funded by DeWorm3 as a staff member of the Benin coordinating team, and by the French Research Institute for Sustainable Development (IRD) through the International Mixed Laboratory LMI CONS_HELM (helminth infections: treatments and consequences on health and development in the South).<br>https://www.ird.fr/benin/partenariat
**Competing Interests**	The authors have declared that no competing interests exist.

Powered by Editorial Manager® and ProduXion Manager® from Aries Systems Corporation

Use the instructions below to enter a competing interest statement for this submission. On behalf of all authors, disclose any competing interests that could be perceived to bias this work—acknowledging all financial support and any other relevant financial or non-financial competing interests.<br><br>This statement **will appear in the published article** if the submission is accepted. Please make sure it is accurate. View published research articles from *PLOS NTDs* for specific examples.<br><br>**NO authors have competing interests**<br><br>Enter: *The authors have declared that no competing interests exist.*<br><br>**Authors with competing interests**<br><br>Enter competing interest details beginning with this statement:<br><br>*I have read the journal's policy and the authors of this manuscript have the following competing interests: [insert competing interests here]*<br><br>* typeset	
**Data Availability**<br><br>Authors are required to make all data underlying the findings described fully available, without restriction, and from the	No - some restrictions will apply

time of publication. PLOS allows rare exceptions to address legal and ethical concerns. See the PLOS Data Policy and FAQ for detailed information.<br><br>A Data Availability Statement describing where the data can be found is required at submission. Your answers to this question constitute the Data Availability Statement and **will be published in the article**, if accepted.<br><br>**Important:** Stating 'data available on request from the author' is not sufficient. If your data are only available upon request, select 'No' for the first question and explain your exceptional situation in the text box.<br><br>Do the authors confirm that all data underlying the findings described in their manuscript are fully available without restriction?	
**Describe where the data may be found in full sentences. If you are copying our sample text, replace any instances of XXX with the appropriate details.**<br><br>• If the data are **held or will be held in a public repository**, include URLs, accession numbers or DOIs. If this information will only be available after acceptance, indicate this by ticking the box below. For example: *All XXX files are available from the XXX database (accession number(s) XXX, XXX.*).<br>• If the data are all contained **within the manuscript and/or Supporting Information files**, enter the following: *All relevant data are within the manuscript and its Supporting Information files.*	Data cannot be shared publicly because the study remains blinded. to outcome data. Data are available from the DeWorm3 Institutional Data Access Committee (contact via Barbra Richardson, barbrar@uw.edu) for researchers who meet the criteria for access to these data.

Powered by Editorial Manager® and ProduXion Manager® from Aries Systems Corporation

• If neither of these applies but you are able to provide **details of access elsewhere**, with or without limitations, please do so. For example:<br><br>*Data cannot be shared publicly because of [XXX]. Data are available from the XXX Institutional Data Access / Ethics Committee (contact via XXX) for researchers who meet the criteria for access to confidential data.*<br><br>*The data underlying the results presented in the study are available from (include the name of the third party and contact information or URL).*<br>• This text is appropriate if the data are owned by a third party and authors do not have permission to share the data.<br><br>* typeset	
Additional data availability information:	Tick here if the URLs/accession numbers/DOIs will be available only after acceptance of the manuscript for publication so that we can ensure their inclusion before publication.; Tick here if your circumstances are not covered by the questions above and you need the journal's help to make your data available.

Manuscript

Click here to access/download;Manuscript;Draft DW3 Benin Baseline_Final_ JLW_EA_30.12.2020.docx

Factors associated with soil-transmitted helminth infection in Benin: findings from the DeWorm3 study

Euripide F G A Avokpaho[1,2♠], Parfait Houngbégnon[1], Manfred Accrombessi[1], Eloic Atindegla[1], Elodie Yard[3], Arianna Rubin Means[3,4], Timothy J Littlewood[5], André Garcia[6], Achille Massougbodji[1], Sean Galagan[4], Judd L Walson[3,4], Gilles Cottrell[6], Moudachirou Ibikounlé[1,7♣], Kristjana Hrönn Ásbjörnsdóttir[3,4♣], Adrian J F Luty[6♣]

♠ Corresponding author: Euripide F G A Avokpaho, email: euripideavokpaho@gmail.com

♣ Joint co-last authors

Authors affiliations:

- [1]Institut de Recherche Clinique du Bénin, Abomey-Calavi, Quartier Zopah Lot 641, 04 BP 1114, Benin.
- [2]Université de Paris, 12 Rue de l'École de Médecine, 75006 Paris, France
- [3]DeWorm3, Division of Life Sciences, Natural History Museum, London, United Kingdom.
- [4]Department of Global Health, University of Washington, Seattle, United States.
- [5]Blantyre Institute for Community Outreach, Lions Sight First Eye Hospital, Blantyre, Malawi.
- [6]Université de Paris, MERIT, IRD, F-75006 Paris, France
- [7] Centre de Recherche pour la lutte contre les Maladies Infectieuses Tropicales (CReMIT), Université d'Abomey-Calavi, 01BP526, Cotonou, Bénin

Introduction

Soil-transmitted helminth (STH) infections are among the most common infections worldwide, affecting more than 1.5 billion of the poorest and most marginalized communities globally. (1) The most common STH ~~species of~~ humans ~~include~~ *Ascaris lumbricoides*, *Trichuris trichiura* and the hookworm species, *Necator americanus* and *Ancylostoma duodenale*. STH are transmitted by eggs present in human feces which in turn contaminate soil and water in areas with poor sanitation, conditions often found

in low-resource countries. (2) STH are widely distributed in tropical and subtropical areas, with the greatest numbers occurring in sub-Saharan Africa, the Americas, China and East Asia. (3)

The World Health Organization (WHO) considers STH a public health problem in areas where >1% of the at-risk population has moderate-to-heavy intensity infection – as measured by number of eggs per gram of stool diagnosed by stool examination (4). These moderate to high intensity helminth infections are associated with poor cognitive and motor outcomes in infants, as well as with anemia. (5–9) Pre-school children (PSAC), school age children (SAC) and women of reproductive age (WRA), including adolescent girls, pregnant women, lactating women, and non-pregnant and non-lactating women living in endemic areas, are at highest risk of morbidity due to STH. Clear policy and guidance are essential to support country-level efforts to expand routine deworming of WRA, and recent WHO publications have provided the necessary policy framework. (2,3)

The WHO Neglected Tropical Disease (NTD) Roadmap and London Declaration have accelerated progress toward eliminating selected NTDs, including lymphatic filariasis and onchocerciasis, and formalized long-term disease-specific goals for other NTDs. (10) Global interest is shifting towards an elimination strategy for other NTDs, including the possibility of breaking the transmission of STH through community-wide mass drug administration (MDA). (11)

In Benin, all major STH (hookworm sp., *A. lumbricoides* and *T. trichiura*) are a recognized public health problem, with more than 50% of districts requiring MDA based on the results of a recent national mapping exercise that sampled stool from SAC. (12,13) In Comé District, this recent national mapping showed a prevalence of STH in school-aged children of 20%, despite multiple rounds of school-based MDA with albendazole in 2015 (coverage 59%), 2016 (coverage 78%) and 2017 (coverage 83%). (13)

In 2017, the DeWorm3 project (ClinicalTrials.gov Identifier NCT03014167) was initiated in Benin, and, in parallel, in India and Malawi. Using a cluster randomized controlled study design, the primary objective of the project is to determine whether

the provision of an enhanced (twice yearly) level of high-coverage MDA, targeting all age groups in a whole community over a 3-year period, can interrupt transmission of STH (11). Here we report analyses of baseline data from a longitudinal monitoring cohort randomly selected from the whole population involved in the trial in order to determine the demographic and other parameters potentially associated with the STH infections detected by microscopy using a standard Kato-Katz procedure.

Materials and Methods

Study area and population

The DeWorm3 trial in Benin is being conducted in the district of Comé. The study site selection was based on criteria reported ~~previously~~. (14) Comé is located 70 km west of Cotonou in the Mono department, at latitude 6°24′N and longitude 1°53′E. The district covers an area of 153 km^2 with a population estimated at 79,989 inhabitants in the census of 2012~~, with an~~ estimated yearly growth rate of 2.07%. (1[illegible]) The district has five sub-districts (Central Comé, Akodéha, Oumako, Agatogbo and Ouèdèmè-Pedah) subdivided into 52 villages/areas or neighborhoods. The climate is sub-equatorial, tropical, alternating between two rainy seasons (April to July and September to November) and two dry seasons (December to March and August). Rainfall varies between 900 and 1,200 mm per year.

Study design

The protocol and aims of the DeWorm3 study have been published elsewhere. (11) A baseline census was conducted from January 8th to February 9^{th,} 2018 followed by cluster demarcation. The geospatial locations of all households were mapped using ArcGIS (Redlands, CA), and the study area was divided into 40 clusters with between 1,650 and 4,000 residents per cluster. From March 6th to April 5th, 2018, 6000 individuals (150 individuals ~~by~~ cluster) were randomly selected to constitute a longitudinal monitoring cohort (LMC) participating in annual follow-up STH infection surveys over 5 years. The LMC was selected from the censused population using stratified random sampling of PSAC aged 1-4 years old, SAC aged 5-14 years old and adults aged 15 years old and above, at a ratio of 1:1:3. A sampling list of 150 individuals (i.e. 30 PSAC, 30 SAC and 90 adults) was initially generated and backup lists of 75

individuals were issued to replace participants who could not be located or refused to participate. LMC participants were interviewed and completed a more in-depth assessment of individual-level STH risk factors, including a survey of self-reported WASH access and use, history of deworming, and direct observation of WASH facilities and participants' use of footwear. Individuals participating in the LMC agreed to provide stool samples for immediate analysis using the Kato-Katz method (16) ~~annually for the duration of the study.~~

Data collection

Kato-Katz data

Stool samples from LMC participants were collected by study staff and delivered to the laboratory within one hour. Samples were screened using the Kato-Katz technique, with results for each type of helminth (*A. lumbricoides*, *T. trichiura* and hookworm: *A. duodenale*/*N. americanus*) reported in eggs per gram (epg). Two slides were prepared from each sample, and each slide was examined by two experienced lab technicians. A subset of 10% of slides was randomly selected for quality assurance by a laboratory manager. Prevalence was calculated both for individual STH types and cumulatively according to the following formulas:

- The prevalence per STH type:

$$p = \frac{\text{Number of samples where at least one egg of STH species is found}}{\text{total number of samples examined}} \text{ x } 100$$

- The cumulative STH prevalence:

$$p = \frac{\text{Number of positive samples for one, two or three STH species}}{\text{total number of samples examined}} \text{ x } 100$$

In cases of co-infection, the sample was counted to calculate the cumulative prevalence, and prevalence and intensity assessed separately for each species. The parasite intensity was calculated from a Kato-Katz smear made with 41.7 mg of stool, by multiplying the egg count from the slide by a factor of 24 (24 x 41.7 mg ≈ 1 g) to get the number of eggs per gram of stool (EPG).

Outcomes

The primary outcomes were individual-level infection status for each STH type (positive / negative) and intensity of infection in ~~eggs per gram (epg)~~.

Variables

Individual factors (including age, gender, history of deworming during the past year and shoe wearing behavior), household factors (including highest educational level achieved, head of household occupation, household asset index, urbanization), water sanitation and hygiene (WASH) factors (household water service, household sanitation, household hand washing facility) and environmental factors (elevation, soil sand fraction, soil acidity at average depth (0-5-15 cm), MODIS daytime land surface temperature mean for 2018 (°Celsius), MODIS Enhanced Vegetation Index (EVI) mean for 2018, MODIS normalized difference vegetation index (NDVI) mean for 2018, aridity index) were collected or constructed based on existing data.

Water, Sanitation and Hygiene variables: Water sources and sanitation facilities reported were grouped and categorized according to the 2017 WHO/UNICEF Joint Monitoring Program (JMP) methodology (none, improved, unimproved, limited or basic). (17) Improved drinking water sources are those that have the potential to deliver safe water by nature of their design and construction, while improved sanitation facilities are those designed to hygienically separate excreta from human contact. (18) Distance to the closest water source and sharing status for sanitation were also collected.

Asset index: An asset index was compiled using principal components analysis. The procedure described by the Demographics and Health Survey *(Steps to constructing the new DHS Wealth Index)*(19) was followed, but factors associated with STH transmission (crowding [residents/room], WASH variables included in the risk factors analysis, and flooring materials) were excluded as they were evaluated separately in the model.

Environmental variables: We examined the association of the following environmental and sociodemographic factors with STH infection: mean enhanced vegetation index

and land surface temperature during the study period; elevation; aridity; soil acidity and sand content; and population density. These environmental, topographical, and sociodemographic measures were extracted for each household using point-based extraction ~~using~~ ArcGIS 10.3 (Environmental Systems Research Institute Inc., Redlands, CA, USA). Data sources and methods have been described ~~previously~~ (20). Estimates of population density were obtained by calculating the number of individuals living within $1km^2$ buffer around each household, which was used to classify areas as high, medium or low population density. Continuous variables were categorized by tertiles for analysis.

Descriptive statistics

Categorical variables were described using proportions and 95% confidence intervals ~~and the~~ continuous variables were described by the median and interquartile ranges. To compare proportions, we used the Chi-square~~,~~ and Cuzick trend tests. Continuous variables were compared using the Student ~~T~~-test and analysis of variance (ANOVA). For each STH species we determined the cluster level prevalence (proportion of individuals infected in the cluster) and cluster level arithmetic mean of individual's egg density per gram of feces. We plotted the cluster level mean egg density against the cluster level prevalence and assessed the strength of the linear relationship using Pearson's correlation coefficient test. Descriptive statistics were generated using Stata® 14.0 (Stata Corp, College Station, Texas).

Factors associated with STH infection

Factors associated with presence and intensity of baseline infection with each STH species were identified using mixed effects models with random effects at the household and cluster levels and exchangeable correlation matrix. For binary infection status, mixed effects logistic regression was used, while for intensity of infection negative binomial mixed effects regression was used.

For the negative binomial regression, the output was the infection intensity ratio (IIR):

$$\text{IIR} = e^{\beta} = e^{[\log(\mu_{x0+1}) - \log(\mu_{x0})]} = e^{[\log(\mu_{x0+1} / \mu_{x0})]}$$

where β is the regression coefficient, μ is the expected intensity of infection (~~epg~~) and the subscripts represent where the predictor variable, say x, is evaluated at x_0 and x_{0+1}

(implying a one unit change in the predictor variable x). The IIR are interpreted as the ratio of expected intensity of infection for a one unit increase in the predictor variable given the other variables are held constant in the model.

All models were adjusted for age and sex. Groups of socio-economic status indicators, environmental factors and WASH factors hypothesized to be associated with infection were proposed *a priori* in the multivariable analysis. For groups of indicators with similar variables, the factor from each group with the lowest Akaike Information Criterion (AIC) in univariate analyses was selected for inclusion in the multivariable model. Models were further simplified by backward stepwise elimination until AIC was no longer further reduced in the adjusted model.

Random effects predicted by the fully adjusted model were compared to those predicted by a model containing only age and sex and the proportion of clustering explained by the explanatory variables was quantified.

Ethics statement

Ethical approval of the DeWorm3 trial protocol was obtained both from the Human Subjects Division at the University of Washington and the National Ethics Committee for Health Research of Benin. (CNERS ethical clearance reference No: 002-2017/MS/DC/SGM/DFR/CNERS-Ministry of Health, Benin). The trial was registered at Clinical Trials.gov NCT03014167. Written consent was obtained from each participant (or participants' parents, when participants were under 18 years of age). For children aged 1-6 years old, verbal assent was obtained and for adolescents aged 7-17 years written assent was obtained. Data were collected electronically using password protected smartphones and was stored in datasets. Although WHO guidelines do not recommend MDA for adults, following the stool analysis any adults (≥15 years of age) in control clusters presenting moderate to heavy intensity STH infection according to WHO definitions (21) or requiring treatment according to local guidelines, were treated with albendazole by study staff.

Results

Descriptive

Based on the census data, 11,979 individuals were selected for participation in three consecutive stages (Stage 1: n=5,979; Stage 2: n=3,000; Stage 3: n=3,000), with the goal to reach 150 individuals in each cluster: 30 PSAC, 30 SAC and 90 adults. Characteristics of the ~~longitudinal monitoring cohort (~~LMC~~)~~ population in comparison to censused population of the DeWorm3 site are presented in Table 1~~, and~~ Fig 1 presents the study flow chart. Individuals selected were listed as living in 9,265 households from which 8,741 were located and visited. In those households 7,045 individuals were present, among whom 6,814 consented to participate in the LMC cohort. Stool samples were collected from 6,153 individuals. The most common reasons for stool samples not being collected were (i) no sample visit documented (319), (ii) sample could not be collected after ~~3~~ visits (n=111), (ii) refusal to provide sample (n=231). As no documented survey could be verified for 14 individuals, Kato-Katz tests performed were confirmed for 6,139 samples comprising 1,184 PSAC (98.7% of 1,200 expected), 1,335 SAC (>100% of 1,200 expected), and 3,620 adults (>100% of 3,600 expected). In total 6,139 tests had two slides read by laboratory technicians. A random subset of Kato-Katz ~~tests~~ was selected for reading by the supervisor and compared against the original readings for quality assurance.

Prevalence of STH

Among the 6139 individuals tested by Kato-Katz, STH infections of any type were found in 324 (5.3%), 199 (3.2%) due to hookworm ~~spp.~~ and 126 (2.0%) due to *Ascaris. Trichuris* was ~~found~~ in ~~just~~ ~~5~~ (0.1%) individuals (Table 2). Six individuals were co-infected with hookworm and *~~Ascaris~~*. Due to the small number of *~~Trichuris~~* infections, only analyses focused on hookworm ~~spp.~~ and *Ascaris* are presented. Among all infections, 258 (79.7%) were light-intensity, 54 (16.7%) moderate-intensity and 12 (3.7%) heavy intensity infections. Hookworm ~~spp. were~~ more prevalent in adults than in SAC or PSAC (4.4% *versus* 2% *versus* 1% respectively, Chi^2, p<0.001). SAC were more ~~frequently~~ infected with *Ascaris* compared to PSAC or adults (3% *versus* 2% *versus* 1.7% respectively, Chi^2, p=0.02) (Fig 2). A higher proportion of males than females was infected with hookworm ~~spp.~~ (4% *versus* 2.6%; p=0.002) and *Ascaris* (2.6% *versus* 1.6%; p=0.004).

Intensity of STH infection

The median egg density for hookworm spp. was 108 eggs per gram (epg) (IQR: 48-312; range: 12-12,960), 3,840 epg for *Ascaris* (IQR: 312-15,180; range: 12-135,084) and 120 epg for *Trichuris* (IQR: 60-468; range: 36-20,124). The intensity of infection was similar in all age groups for hookworm spp. (ANOVA, p=0.22), with a median egg density of 264 epg (IQR: 36-384; range: 12-3,048) in PSAC, 96 epg (IQR: 24-312; range: 12-11,100) in SAC and 108 epg (IQR: 48-288; range: 12-12,960) in adults. We found a difference in intensity of infection with *Ascaris* between age-groups (ANOVA, p=0.005), this difference was between SAC and adults (Bonferroni, p=0.004). Median egg densities were 6,972 epg for PSAC (IQR: 264-26292; range: 12-60000), 7,848 epg for SAC (IQR: 3,714-25,314; range: 84-56,412) and 780 epg for adults (IQR: 36-8,772; range: 12-135084).

Moderate to heavy intensity (MHI) infections were found in 66/6,139 individuals overall (1.1%) amongst whom 10 (0.2%) MHI with hookworm spp., 55 (0.9%) MHI with *Ascaris* and 1 (<0.1%) MHI with *Trichuris* (Table 2). The burden of MHI was greatest in SAC with 2.1% (25/1,184) prevalence of MHI of *Ascaris* (Table S1). 68.2% (45/66) of MHI were found in males (Table S2). MHI were distributed in 15/40 clusters. MHI with hookworm spp. were present in 7/40 clusters, MHI with *Ascaris* in 7/40 clusters and MHI with *Trichuris* in 1 cluster. There were two clusters showing a particularly high burden of *Ascaris*, with respectively 19 (12.7%) and 30 (20%) individuals with MHI with *Ascaris*.

Age- and sex-related prevalence and intensity of STH infection (hookworm and *Ascaris*)

Figs 3 and 4 show the age-infection profile for hookworm spp. and *Ascaris,* respectively. The prevalence of hookworm spp. increased with age in both sexes. The prevalence was similar in males and females among PSAC and SAC but in adults, the prevalence in males was higher than in females except for 50-60 year olds, in whom females were more frequently infected. The intensity of hookworm infection was similar in males and females regardless of age, and was higher in adults than in children. The prevalence of *Ascaris* infection was similar in males and females across all ages, with the period of adolescence and early adulthood (between 10 and 18 years old) corresponding to the period with highest prevalence of *Ascaris* infection in males and

the lowest in females (6% for males *versus* 1% for females). Intensity of *Ascaris* infection followed the same profile as prevalence in both sexes.

Community-level correlation between intensity and prevalence of STH infection

We found a positive linear relationship between STH infection prevalence and the intensity of infection at cluster level in our study population (Fig 5). This correlation was strong for both hookworm spp. (ρ=0.73, p<0.0001) and *Ascaris* (ρ=0.98, p<0.0001).

Factors associated with hookworm infection

The results of univariate analyses of factors associated with hookworm infection prevalence are presented in Table S2. ~~Here,~~ the results of multivariable analyses ~~are presented~~ (Table 3).

At ~~the~~ individual level, PSAC and SAC were significantly less likely to be infected with hookworm ~~spp.~~ than adults (aOR=0.21, 95%CI 0.10-0.44, p< 0.001 and aOR=0.49, 95%CI 0.29-0.83, p=0.008, respectively). Females were also significantly less likely to be infected than males (aOR=0.56, 95%CI 0.38-0.83, p=0.004). Individuals who reported a history of deworming during the past year were significantly less likely to be infected (aOR= 0.45, 95%CI 0.27-0.75, p< 0.002).

Among household factors, the household asset index, a proxy measure for family wealth, showed that individuals in the poorest households had a significantly higher odds of infection than the richest (5th quintile) with a significant dose-response effect (Cuzick test of trend, p<0.001), (First quintile: aOR= 5.03, 95%CI 2.10-12.01, p<0.001, 2nd quintile aOR= 3.62, 95%CI 1.51-8.66, p=0.001 and 3rd quintile aOR= 2.51, 95%CI 1.05-6.00, p=0.02). With respect to occupational exposure, farmers were more likely to be infected with hookworm spp. than others (aOR= 1.79, 95%CI 1.11-2.90, p=0.02). Individuals living in medium population density settings were more likely to be infected than those living in high density settings, (aOR= 2.59, 95%CI 1.25-5.40, p=0.01).

Among WASH factors, household sanitation~~,~~ and ~~especially~~ open defecation~~, was~~ found to be strongly associated with hookworm infection. Individuals using improved

unshared sanitation facilities had half the odds of hookworm infection compared to those defecating outdoors (aOR=0.48, 0.24-0.98, p=0.04).

Factors associated with hookworm infection intensity

Children had a significantly lower intensity of hookworm infection as compared to adults (PSAC: adjusted IIR=0.10, 95%CI 0.03-0.31, p<0.001; SAC: adjusted IIR=0.29, 95%CI 0.12 – 0.72, p=0.01. Females had significantly lower intensity infections than males (adjusted IIR=0.32, 95%CI 0.16–0.64, p=0.001), as did individuals dewormed the year before (adjusted IIR=0.20, 95%CI 0.08–0.48, p<0.001). (Table 3).

At the household level, less educated people (those with no education, primary school or secondary school) had higher intensity infections with hookworm spp. compared to those with university level education (adjusted IIR=40.13, 95%CI 2.47–652.77, p=0.01; adjusted IIR=30.92, 95%CI 1.86–513.88, p=0.02; adjusted IIR=19.34, 95%CI 1.21–308.81, p=0.04, respectively). Being a farmer, living in a house with natural floor material versus man-made floor material, and living in a peri-urban setting were also all factors found to be associated with a significantly increased intensity of hookworm infections (farmer: adjusted IIR=3.94, 95%CI 1.67–9.27, p=0.002; natural floor material: adjusted IIR=0.23, 95%CI 0.05-1.03, p=0.06; peri-urban settings: adjusted IIR=6.18, 95%CI 1.82 - 20.90, p=0.003).

Access to unimproved water available more than 30 minutes away from the house was associated with significantly higher intensity hookworm infection (adjusted IIR= 13.47, 95%CI 1.62–111.55; p=0.02) compared to improved water available less than 30 minutes from the house. Compared to open defecation behavior, using an improved and unshared toilet was associated with significantly lower intensity hookworm infections (adjusted IIR=0.23, 95%CI 0.07 – 0.70, p=0.01). No environmental factor was found to be associated with intensity of hookworm infections in multivariable analyses.

Factors associated with *Ascaris* infection prevalence.

Among the individual factors assessed, SAC (5-14 years) were significantly more likely to be infected with *Ascaris* than adults (aOR= 2.0, 95%CI 1.1-3.6, p=0.01). However, no difference in odds of infection was found between PSAC and adults. Female individuals were less likely to be infected with *Ascaris* than males (aOR= 0.5, 95%CI 0.3-0.9, p= 0.02).

Amongst environmental factors, low soil acidity was significantly associated with increased odds of *Ascaris* infection compared to the highest soil acidity (aOR=4.8, 95%CI 1.8-13.1, p=0.002). Moderate [29.6-31.9°C] and high [31.9; 32.8°C] daytime land surface temperatures were associated with lower odds of infection with *Ascaris* compared to lower temperatures [26.2-29.6°C[(aOR=0.12, 95%CI 0.03-0.44, p=0.001; and aOR=0.17, 95%CI 0.03-0.91, p= 0.04 respectively). The summary of the multivariable analysis with *Ascaris* is presented in Table 4.

Intra-Class Correlation statistics for hookworm and *Ascaris* infection prevalence.

Comparison of the Intra-Class Correlation values between models containing only age and sex (model 1) and the fully adjusted multivariable final model with all the fixed effect covariables (model 2) showed decreased ICC values in the fully adjusted model, considering either level-3 ICC at the cluster level or level-2 ICC at the household-within-cluster level (Table S4).

When only adjusting for age and sex, the prevalence of hookworm infection was correlated between individuals within the same cluster (ICC=0.16, 95%CI 0.10-0.26), and this correlation increased significantly between individuals within the same household and cluster level (ICC=0.58, 95%CI 0.40-0.74). Prevalence of *Ascaris* infection was moderately correlated within the same cluster (ICC=0.54, 95%CI 0.34-0.73), with a small increase within the same household and cluster level (ICC=0.60, 95%CI 0.26-0.76). In this model, household and cluster random effects compose approximately 58% and 60% of the total residual variance for hookworm spp. and *Ascaris* infection prevalence respectively.

In the fully adjusted multivariable final model, we found a correlation of hookworm infection prevalence within the same cluster (ICC=0.03, 95%CI 0.01-0.10), although this correlation increased within the same household and cluster level (ICC=0.39, 95%CI 0.17-0.65). *Ascaris* infection prevalence was moderately correlated between individuals within the same cluster (ICC=0.42, 95%CI 0.23-0.64), and this correlation increased slightly within the same household and cluster level (ICC=0.51, 95%CI 0.26-0.76). We estimated that household and cluster random effects compose approximately 39% and 51% of the total residual variance of hookworm and *Ascaris* infection prevalence, respectively, in the fully adjusted model.

Discussion

We observed a relatively low prevalence of STH in this region of Benin. Hookworm spp. were the most prevalent infections, and were more prevalent in adults, while *Ascaris* was more prevalent in children. Females were generally less infected than males across all ages. Females, children, those dewormed during the previous year and those using improved unshared sanitation facilities had lower odds of hookworm infections, while being a farmer, living in peri-urban settings versus urban and being poor was associated with a higher odds of hookworm infection. In addition to those factors, the intensity of hookworm infection was also decreased if an improved water source was available at less than 30 minutes distance.

Since 2013, the Ministry of Health in Benin has focused its efforts on developing and implementing strategies for the control of five NTDs considered to be of highest priority, namely trachoma, onchocerciasis, lymphatic filariasis, schistosomiasis and soil-transmitted helminths. Those efforts were bolstered markedly through the ENVISION program (22), a USAID-funded initiative that ran from 2013 through 2019 in Benin. A nationwide STH prevalence survey was completed in 2015, that reported 20% prevalence (13) in school-aged children in Comé district. Following that national mapping effort, 3 rounds of school-based MDA with albendazole were undertaken according to the recommendations of WHO, i.e. primarily targeting school-age (SAC) and pre-school age children (PSAC) for either once or twice yearly treatment as a function of the estimated prevalence of infection in any given district. (23) Coverage of

SAC with school MDA between 2015 and 2017 was estimated between 59% and 83%. Albendazole and/or Mebendazole are also distributed in health facilities and to pregnant women during routine antenatal care starting from the 2nd trimester of pregnancy. (24) In the context described, we sought to better understand patterns of STH infection in order to move towards the elimination STH as a public health problem, by reaching a prevalence of STH less than 1%, as prescribed by the WHO Neglected Tropical Disease (NTD) Roadmap and London Declaration on NTD. (10,23)

When focusing on the at-risk population of SAC, the prevalence of STH infection found in the current study is lower than that reported in the same district using the same diagnostic technique in 2015 during the national mapping exercise (5.2% versus 20.0% respectively, p<0.001). That survey was conducted with a total of 250 stool samples from SAC collected from schools located in 5 rural villages. (13) The prevalence of infections with *Ascaris* (3.0% *versus* 15.6% respectively, p<0.001) or *Trichuris* (0.15% in 2018 *versus* 4.8% in 2015, p<0.001) decreased while the decrease in prevalence of hookworm spp. in SAC was less marked (2.0% in 2018 versus 4.0% in 2015, p=0.054) compared to the findings of the national STH mapping 3 years earlier. (13) The decline in STH prevalence in the study area might be related to differences in sampling, as the current study was conducted in the community instead of in schools, with more than 6,000 stools randomly selected from three age groups (PSAC, SAC and adults). (11) STH prevalence estimates can vary depending on the sampling strategies used. (25) The reasons for the observed variations of prevalence between hookworm spp. and other STH species in SAC could also be that STH rate of reinfection post-treatment varies across species, with a faster reinfection with *Ascaris* than hookworm spp. A systematic review of helminth reinfection at 3, 6, and 12 months (95% CI), after drug treatment shows that *Ascaris* prevalence reached 26% (16-43%), 68% (60-76%) and 94% (88-100%) of pretreatment levels, respectively and for hookworm spp., 30% (26-34%), 55% (34-87%), and 57% (49-67%). (26) These results may also be partly explained by the fact that the current STH program does not include adults. The suggestion that hookworm spp. prevalence only decreased slightly between 2015 and 2018, may be due to the persistent untreated adult reservoir in which hookworm spp. are most common. Data from several worm expulsion studies show that the proportion of hookworms harbored by adults ranged from 70 to 85%, (27–32) and a reinfection–infection study in Indonesia show that adults have higher reinfection rates with

hookworm spp. than children. (33) Children cleared of hookworms through annual school de-worming could easily be re-infected at home through contact with adult members of their households.

Hookworm prevalence was higher in adults while *Ascaris* prevalence was higher in children. One explanation of these findings might be helminth species transmission modes. (34) The three species of STH (*A. lumbricoides*, *T. trichiura*, hookworm spp.) have relatively similar cycles involving the presence of adult worms in the intestine, however, the main mode of transmission of *Ascaris* and *Trichuris* is through contaminated food and water (parasite egg ingestion) whereas hookworm spp. are mainly transmitted by skin penetration, although they can be transmitted by ingestion. (35,36) The eggs of *Ascaris* and *Trichuris* are found in soil contaminated by human feces or in uncooked food contaminated by soil containing eggs of the worm. A person becomes infected after accidentally swallowing the fertile eggs. Children may be more likely to be infected with *Ascaris* because they are more likely to put their contaminated fingers in their mouths after playing in contaminated soil. (37) Unlike *Ascaris* infection, which declines in prevalence with age, hookworm infects all ages throughout life with prevalence increasing in adults. (27)

Community-level prevalence and the arithmetic mean of infection intensity were significantly correlated for all STH infections in our study, with a strong prevalence-intensity correlation for infection with hookworm and *Ascaris*. Similar trends were recently found in Kenya for hookworm spp. and *Trichuris.* (20) At the individual level, prevalence and intensity of hookworm infection followed the same trend. Markers of poverty and exposure to environmental sources of STH infection, including being a farmer, lack of improved or private sanitation facilities, low income, poor access to water, no or limited education, or living in a house with natural floor material were all associated with a higher prevalence or intensity of hookworm infection. These findings are linked with the mode of hookworm spp. transmission, which is direct either by ingestion (for *A. duodenale*) or by skin penetration (both *N. americanus* and *A. duodenale*) of infective larval stages living in the soil. (38,39) These findings are consistent with the results of a recent study in Kenya where there was a strong association between hookworm infection prevalence and intensity and socio-economic status, with those in the poorest households having the heaviest infections and highest prevalence, and wealthier individuals having the lightest intensity and reduced odds of

infection. (20) Globally, a negative correlation between hookworm infections and income level is demonstrated in cross-country comparisons. (40–42) Moderate population density, corresponding to a peri-urban environment, was also associated with both high prevalence and heavy intensity of hookworm infection when compared to the higher population density observed in urban environments. (43)

Although we found no association between hookworm prevalence and water source, quality of water seems to affect intensity of hookworm infection. Heavier intensity infections were found in participants with access only to unimproved water, such as unprotected wells, unprotected springs and surface water available at more than 30 minutes from the house. In a school survey in Togo, unimproved drinking water was associated with higher odds and intensity of hookworm. (44) Malaysian children with access to piped water were less infected with hookworm. (45) However, other researchers have found no statistically significant associations between piped water access and hookworm infection (46,47). We did not find any association between WASH variables and either prevalence or intensity of *Ascaris* infection. However, there is evidence that integrated water, sanitation and hand hygiene intervention, treatment of water with chlorine (48), drinking piped water, as well as hand washing before eating and after defecating reduce the odds of *Ascaris* infection. (49)

This study had a number of strengths, including the large population size, completeness and quality of data and the high level of quality control for Kato-Katz diagnosis, with double reading by the lab technicians of the whole sample with an additional control of a subset of samples by a senior skilled parasitologist. However, this study does has some limitations. First, the STH prevalence in Come hides inter- and intra-specific variations between clusters that will be developed in further analyses once the parent study is unblinded and we have access to those results. It was also necessary to use a staged approach to sampling in order achieve the required number of participants who consented to participate in the longitudinal monitoring cohort, which may have limited its representativeness. Another possible limitation is the reliance on Kato-Katz to detect STH. Kato-Katz is poorly sensitive, particularly for low intensity infections and can be affected by storage and processing time and methods.(50,51)

Future analyses using qPCR-based methods will allow for more sensitive detection of of STH in stool.

Conclusion

This analysis of the DeWorm3 baseline study data shows that hookworm ~~spp. are~~ the predominant STH in Comé, with a persistent reservoir in adults. This infection reservoir is not addressed by current school-based MDA control measures. These data suggest that community-based MDA may help eliminate STH as a public health problem. Improved unshared sanitation and access to improved water sources are associated with lower prevalence and/or intensity of hookworm infection. Programmatic efforts should pay particular attention to farmers and populations living in poverty in urban, rural and peri-urban environments. The DeWorm3 trial (2017-2022) will determine the feasibility of STH transmission interruption through community-wide MDA given twice-a-year for three years in this setting ~~and combined~~ with these results will inform programmatic and policy decisions to improve efforts to eliminate morbidity and infection due to these pervasive infections.

Declaration of competing interest:

The DeWorm3 study is funded by Bill and Melinda Gates foundation. EA is a PhD candidate at the University of Paris. His research is funded by DeWorm3 as a staff member of the Benin coordinating team, and by the French Research Institute for Sustainable Development (IRD) through the International Mixed Laboratory LMI CONS_HELM (helminth infections: treatments and consequences on health and development in the South).

Authors' contribution:

EFGAA, PH, MA, EA, GC, MI, AJFL, EY contributed to data collection,
EFGAA performed the statistical analysis and wrote the first draft of the manuscript,

KHA designed the article statistical methodology and reviewed the analysis and entire article draft,

PH add inputs to the statistical analysis,

KHA, ARM, JLW, TJL, SG, designed the Deworm3 clinical trial

EFGAA, MA, KHA, AJFL, MI, AG, JLW reviewed the article draft and the final version.

Funding: Bill and Melinda Gates foundation

Sponsor: Natural History Museum, United Kingdom

Collaborators:

Bill and Melinda Gates Foundation

University of Washington

Imperial College London

London School of Hygiene and Tropical Medicine

Institut de Recherche Clinique du Bénin

Institut de Recherche pour le Développement

Acknowledgements

Thanks to Kate Halliday for sharing a Stata code to illustrate the age-infection profile for hookworm and *Ascaris*. We are particularly grateful to William Oswald, David Kennedy and Mira Emmanuel-Fabula for their invaluable contributions to data collection and quality.

We thank Dr Batcho and his staff of the National NTD Program (PNLMT), and finally we are grateful to all Comé district population, administrative and health authorities for their support in the success of this study.

References

1. Hotez P, Ottesen E, Fenwick A, Molyneux D. The neglected tropical diseases: the ancient afflictions of stigma and poverty and the prospects for their control and elimination. Adv Exp Med Biol. 2006;582:23–33.

2. Soil-transmitted helminth infections [Internet]. [cited 2019 Nov 3]. Available from: https://www.who.int/news-room/fact-sheets/detail/soil-transmitted-helminth-infections

3. WHO | 12th meeting of the Strategic and Technical Advisory Group for Neglected Tropical Diseases [Internet]. [cited 2020 Apr 4]. Available from: https://www.who.int/neglected_diseases/events/twelfth_stag/en/

4. Soil-transmitted helminthiases : eliminating as public health problem soil-transmitted helminthiases in children : progress report 2001-2010 and strategic plan 2011-2020 [Internet]. [cited 2020 Apr 24]. Available from: https://apps.who.int/iris/handle/10665/44804

5. Mpairwe H, Tweyongyere R, Elliott A. Pregnancy and helminth infections. Parasite Immunol. 2014 Aug;36(8):328–37.

6. Mireku MO, Boivin MJ, Davidson LL, Ouédraogo S, Koura GK, Alao MJ, et al. Impact of Helminth Infection during Pregnancy on Cognitive and Motor Functions of One-Year-Old Children. Kang G, editor. PLoS Negl Trop Dis. 2015 Mar 10;9(3):e0003463.

7. Gall S, Müller I, Walter C, Seelig H, Steenkamp L, Pühse U, et al. Associations between selective attention and soil-transmitted helminth infections, socioeconomic status, and physical fitness in disadvantaged children in Port Elizabeth, South Africa: An observational study. Flisser A, editor. PLoS Negl Trop Dis. 2017 May 8;11(5):e0005573.

8. Larocque R, Casapia M, Gotuzzo E, Gyorkos TW. RELATIONSHIP BETWEEN INTENSITY OF SOIL-TRANSMITTED HELMINTH INFECTIONS AND ANEMIA DURING PREGNANCY. Am J Trop Med Hyg. 2005 Oct 1;73(4):783–9.

9. Welch VA, Ghogomu E, Hossain A, Awasthi S, Bhutta ZA, Cumberbatch C, et al. Mass deworming to improve developmental health and wellbeing of children in low-income and middle-income countries: a systematic review and network meta-analysis. Lancet Glob Health. 2018 May 22;5:e40-50.

10. London Declaration on Neglected Tropical Diseases | Uniting to Combat NTDs [Internet]. [cited 2020 Apr 5]. Available from: https://unitingtocombatntds.org/london-declaration-neglected-tropical-diseases/

11. Ásbjörnsdóttir KH, Ajjampur SSR, Anderson RM, Bailey R, Gardiner I, Halliday KE, et al. Assessing the feasibility of interrupting the transmission of soil-transmitted helminths through mass drug administration: The DeWorm3 cluster randomized trial protocol. Garba A, editor. PLoS Negl Trop Dis. 2018 Jan 18;12(1):e0006166.

12. Boko PM, Ibikounle M, Onzo-Aboki A, Tougoue J-J, Sissinto Y, Batcho W, et al. Schistosomiasis and Soil Transmitted Helminths Distribution in Benin: A Baseline Prevalence Survey in 30 Districts. Serrano Ferron E, editor. PLOS ONE. 2016 Sep 19;11(9):e0162798.

13. Ibikounlé M, Onzo-Aboki A, Doritchamou J, Tougoué J-J, Boko PM, Savassi BS, et al. Results of the first mapping of soil-transmitted helminths in Benin: Evidence of countrywide hookworm predominance. Aksoy S, editor. PLoS Negl Trop Dis. 2018 Mar 1;12(3):e0006241.

14. Team TDT. Baseline patterns of infection in regions of Benin, Malawi and India seeking to interrupt transmission of soil transmitted helminths (STH) in the DeWorm3 trial. PLoS Negl Trop Dis. 2020 Nov 2;14(11):e0008771.

15. EDS_2012_Rapport_final-11-15-2013.pdf [Internet]. [cited 2020 Apr 5]. Available from: https://www.insae-bj.org/images/docs/insae-statistiques/enquetes-recensements/EDS/2011-2012/EDS_2012_Rapport_final-11-15-2013.pdf

16. Katz N, Chaves A, Pellegrino J. A simple device for quantitative stool thick-smear technique in Schistosomiasis mansoni. Rev Inst Med Trop Sao Paulo. 1972 Dec;14(6):397–400.

17. Methods | JMP [Internet]. [cited 2020 Oct 4]. Available from: https://washdata.org/monitoring/methods

18. Home | JMP [Internet]. [cited 2020 Jan 19]. Available from: https://washdata.org/

19. Shea O. Rutstein, DHS Asset index calculation - Recherche Google [Internet]. [cited 2019 Dec 31]. Available from: https://www.google.bj/search?sxsrf=ACYBGNQVqWgdnooBO2f7bESKoDpxgi_Eig%3A1577831986052&ei=Ms4LXu7pAoeiUsLIjKAD&q=Shea+O.+Rutstein%2C+DHS+Asset+index+calculation&oq=Shea+O.+Rutstein%2C+DHS+Asset+index+calculation&gs_l=psy-ab.3..33i160.142205.151213..151495...0.0..0.294.7369.2-31......0....1..gws-wiz.......35i39j33i21.ZigUpaQrJxY&ved=0ahUKEwjuyqyY-uDmAhUHkRQKHUIkAzQQ4dUDCAs&uact=5

20. Halliday KE, Oswald WE, Mcharo C, Beaumont E, Gichuki PM, Kepha S, et al. Community-level epidemiology of soil-transmitted helminths in the context of school-based deworming: Baseline results of a cluster randomised trial on the coast of Kenya. Garba A, editor. PLoS Negl Trop Dis. 2019 Aug 9;13(8):e0007427.

21. WHO Expert Committee. Prevention and control of schistosomiasis and soil-transmitted helminthiasis. World Health Organ Tech Rep Ser. 2002;912:i–vi, 1–57, back cover.

22. ENVISION | a world free of NTDs [Internet]. [cited 2020 Apr 5]. Available from: http://www.ntdenvision.org/

23. NTD_RoadMap_2012_Fullversion.pdf [Internet]. [cited 2020 Apr 5]. Available from: https://www.who.int/neglected_diseases/NTD_RoadMap_2012_Fullversion.pdf

24. Weltgesundheitsorganisation, editor. Preventive chemotherapy in human helminthiasis: coordinated use of anthelminthic drugs in control interventions ; a manual for health professionals and programme managers. Geneva: World Health Organization; 2006. 62 p.

25. Giardina F, Coffeng LE, Farrell SH, Vegvari C, Werkman M, Truscott JE, et al. Sampling strategies for monitoring and evaluation of morbidity targets for soil-transmitted helminths. Lustigman S, editor. PLoS Negl Trop Dis. 2019 Jun 26;13(6):e0007514.

26. Jia T-W, Melville S, Utzinger J, King CH, Zhou X-N. Soil-transmitted helminth reinfection after drug treatment: a systematic review and meta-analysis. PLoS Negl Trop Dis. 2012 Jan 1;6(5):e1621.

27. Turner HC, Truscott JE, Bettis AA, Shuford KV, Dunn JC, Hollingsworth TD, et al. An economic evaluation of expanding hookworm control strategies to target the whole community. Parasit

Vectors [Internet]. 2015 Nov 5 [cited 2020 Apr 25];8. Available from: https://www.ncbi.nlm.nih.gov/pmc/articles/PMC4635541/

28. Ye XP, Wu ZX, Sun FH. The population biology and control of Necator americanus in a village community in south-eastern China. Ann Trop Med Parasitol. 1994 Dec;88(6):635–43.

29. Bradley M, Chandiwana SK, Bundy DA, Medley GF. The epidemiology and population biology of Necator americanus infection in a rural community in Zimbabwe. Trans R Soc Trop Med Hyg. 1992 Feb;86(1):73–6.

30. Haswell-Elkins MR, Elkins DB, Manjula K, Michael E, Anderson RM. An investigation of hookworm infection and reinfection following mass anthelmintic treatment in the south Indian fishing community of Vairavankuppam. Parasitology. 1988 Jun;96 (Pt 3):565–77.

31. Pritchard DI, Quinnell RJ, Slater AF, McKean PG, Dale DD, Raiko A, et al. Epidemiology and immunology of Necator americanus infection in a community in Papua New Guinea: humoral responses to excretory-secretory and cuticular collagen antigens. Parasitology. 1990 Apr;100 Pt 2:317–26.

32. Anderson RM, Schad GA. Hookworm burdens and faecal egg counts: an analysis of the biological basis of variation. Trans R Soc Trop Med Hyg. 1985;79(6):812–25.

33. Soeripto N. Reinfection and infection rates of soil-transmitted-helminths in Kemiri Sewu, Yogyakarta, Indonesia. Southeast Asian J Trop Med Public Health. 1991 Jun;22(2):216–21.

34. Truscott JE, Turner HC, Farrell SH, Anderson RM. Soil-Transmitted Helminths: Mathematical Models of Transmission, the Impact of Mass Drug Administration and Transmission Elimination Criteria. Adv Parasitol. 2016;94:133–98.

35. Hookworm. - PubMed - NCBI [Internet]. [cited 2019 Nov 3]. Available from: https://www.ncbi.nlm.nih.gov/pubmed/?term=Banwell+JG%2C+Schad+GA.+(1978)+Hookworm.+Clin+Gastroenterol.+7%3A129%E2%80%9356.

36. Prevention C-C for DC and. CDC - Hookworm [Internet]. 2019 [cited 2020 Apr 25]. Available from: https://www.cdc.gov/parasites/hookworm/index.html

37. WHO | Water related diseases [Internet]. WHO. World Health Organization; [cited 2020 Apr 25]. Available from: http://www.who.int/water_sanitation_health/diseases-risks/diseases/ascariasis/en/

38. Hoagland KE, Schad GA. Necator americanus and Ancylostoma duodenale: life history parameters and epidemiological implications of two sympatric hookworms of humans. Exp Parasitol. 1978 Feb;44(1):36–49.

39. Banwell JG, Schad GA. Hookworm. Clin Gastroenterol. 1978 Jan;7(1):129–56.

40. de Silva NR, Brooker S, Hotez PJ, Montresor A, Engels D, Savioli L. Soil-transmitted helminth infections: updating the global picture. Trends Parasitol. 2003 Dec 1;19(12):547–51.

41. Brooker S, Bethony J, Hotez PJ. Human Hookworm Infection in the 21st Century. Adv Parasitol. 2004;58:197–288.

42. Fenghua S, Zhongxing W, Yixing Q, Hangqun C, Haichou X, Hainan R, et al. Epidemiology of human intestinal nematode infections in Wujiang and Pizhou counties, Jiangsu Province, China. Southeast Asian J Trop Med Public Health. 1998 Sep;29(3):605–10.

43. Yapi RB, Chammartin F, Hürlimann E, Houngbedji CA, N'Dri PB, Silué KD, et al. Bayesian risk profiling of soil-transmitted helminth infections and estimates of preventive chemotherapy for school-aged children in Côte d'Ivoire. Parasit Vectors [Internet]. 2016 Mar 21 [cited 2019 Nov 12];9. Available from: https://www.ncbi.nlm.nih.gov/pmc/articles/PMC4802658/

44. Baker JM, Trinies V, Bronzan RN, Dorkenoo AM, Garn JV, Sognikin S, et al. The associations between water and sanitation and hookworm infection using cross-sectional data from Togo's national deworming program. PLoS Negl Trop Dis [Internet]. 2018 Mar 28 [cited 2019 Nov 12];12(3). Available from: https://www.ncbi.nlm.nih.gov/pmc/articles/PMC5902041/

45. Nasr NA, Al-Mekhlafi HM, Ahmed A, Roslan M, Bulgiba A. Towards an effective control programme of soil-transmitted helminth infections among Orang Asli in rural Malaysia. Part 1: Prevalence and associated key factors. Parasit Vectors. 2013;6(1):27.

46. Cundill B, Alexander N, Bethony JM, Diemert D, Pullan RL, Brooker S. Rates and intensity of re-infection with human helminths after treatment and the influence of individual, household, and environmental factors in a Brazilian community. Parasitology. 2011 Sep;138(11):1406–16.

47. Kounnavong S, Vonglokham M, Houamboun K, Odermatt P, Boupha B. Soil-transmitted Helminth Infections and Risk Factors in Preschool Children in Southern Rural Lao People's Democratic Republic. Trans R Soc Trop Med Hyg. 105(3):160–3.

48. Pickering AJ, Njenga SM, Steinbaum L, Swarthout J, Lin A, Arnold BF, et al. Effects of single and integrated water, sanitation, handwashing, and nutrition interventions on child soil-transmitted helminth and Giardia infections: A cluster-randomized controlled trial in rural Kenya. PLoS Med [Internet]. 2019 Jun 26 [cited 2019 Nov 12];16(6). Available from: https://www.ncbi.nlm.nih.gov/pmc/articles/PMC6594579/

49. Strunz EC, Addiss DG, Stocks ME, Ogden S, Utzinger J, Freeman MC. Water, Sanitation, Hygiene, and Soil-Transmitted Helminth Infection: A Systematic Review and Meta-Analysis. PLoS Med [Internet]. 2014 Mar 25 [cited 2020 Jan 10];11(3). Available from: https://www.ncbi.nlm.nih.gov/pmc/articles/PMC3965411/

50. Bärenbold O, Raso G, Coulibaly JT, N'Goran EK, Utzinger J, Vounatsou P. Estimating sensitivity of the Kato-Katz technique for the diagnosis of Schistosoma mansoni and hookworm in relation to infection intensity. PLoS Negl Trop Dis [Internet]. 2017 Oct 4 [cited 2020 Oct 24];11(10). Available from: https://www.ncbi.nlm.nih.gov/pmc/articles/PMC5643140/

51. Nikolay B, Brooker SJ, Pullan RL. Sensitivity of diagnostic tests for human soil-transmitted helminth infections: a meta-analysis in the absence of a true gold standard. Int J Parasitol. 2014 Oct 1;44(11):765–74.

Click here to access/download;Figure;Fig1.tif

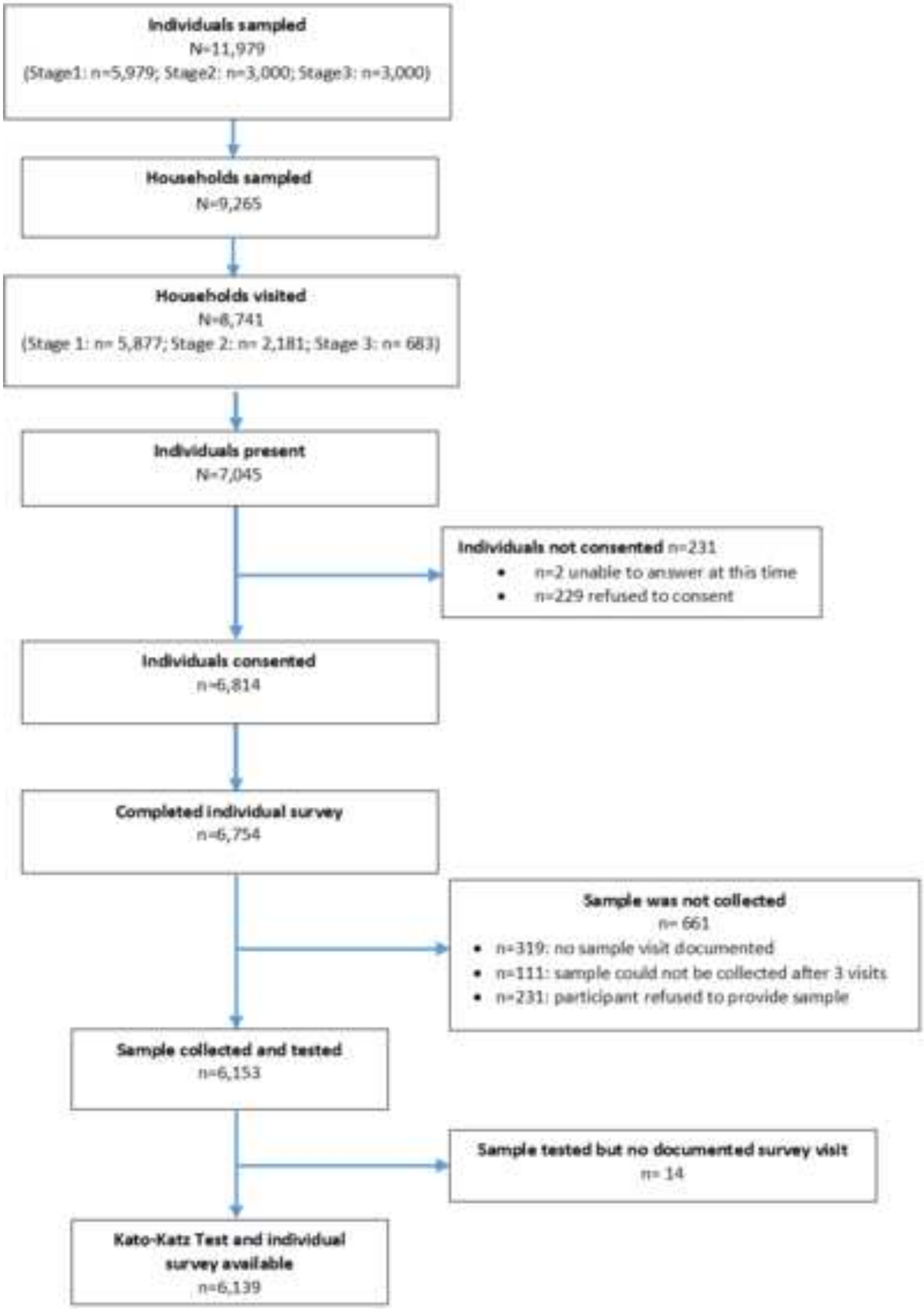

Fig 1: Flow diagram of stool sample collection for Benin site DeWorm3 baseline prevalence survey in Comé

Figure 2: STH unweighted prevalence across age-groups

Click here to access/download;Figure;Fig2.tif

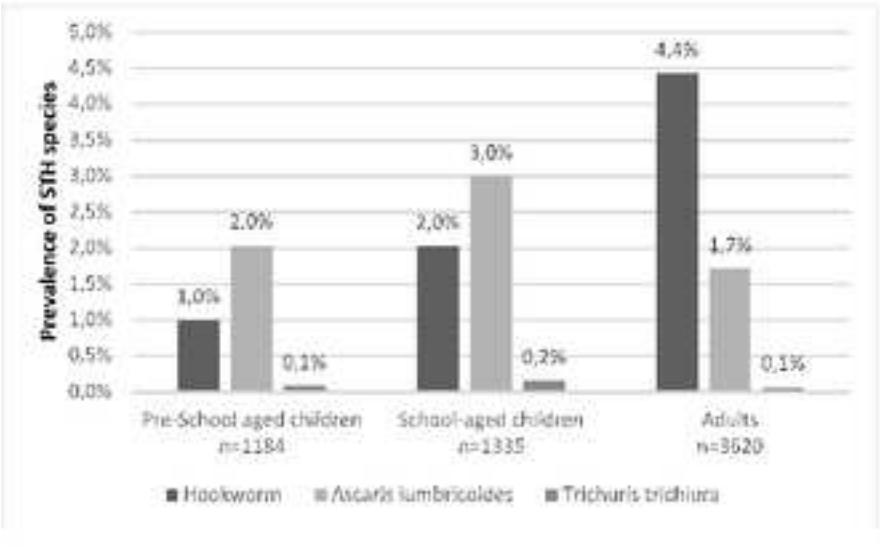

Fig 2: STH unweighted prevalence across age-groups

Click here to access/download;Figure;Fig3.tif

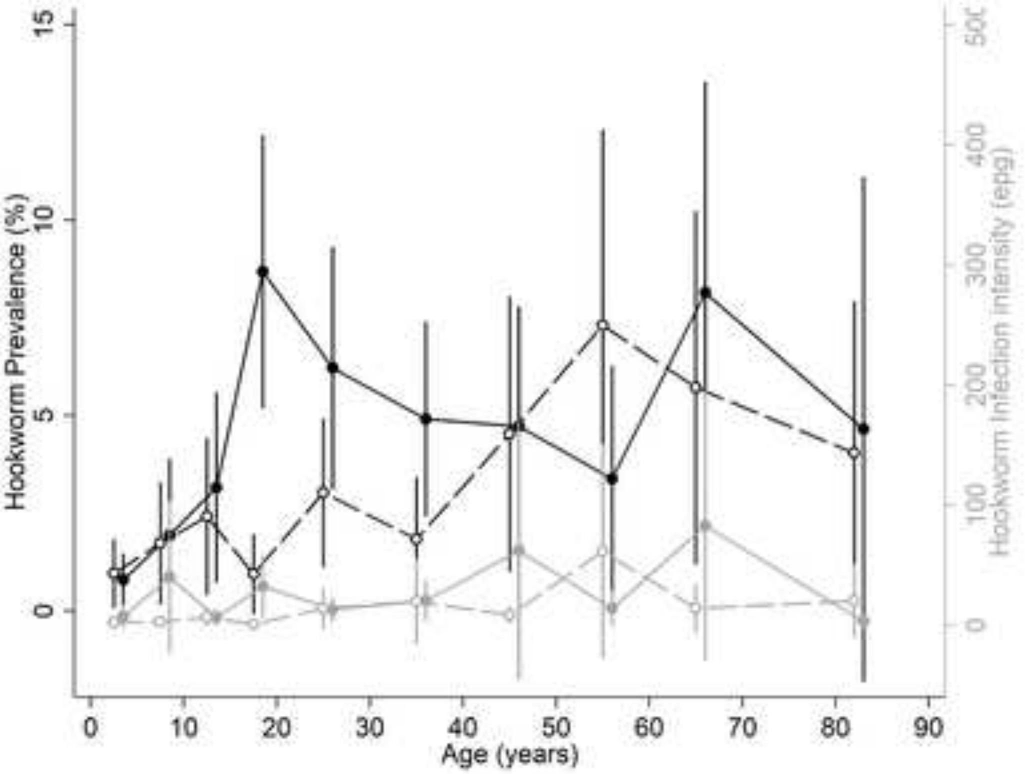

Fig 3. Age-infection profiles for hookworm. (A) Prevalence (black lines) and intensity (grey lines) of hookworm infection by age for males (solid line and circles) and females (dashed lines and empty circles). Vertical bars represent confidence intervals.

Fig 4: Age-infection profiles for Ascaris. (A) Prevalence (black lines) and intensity (grey lines) of Ascaris infection by age for

Click here to access/download;Figure;Fig4.tif

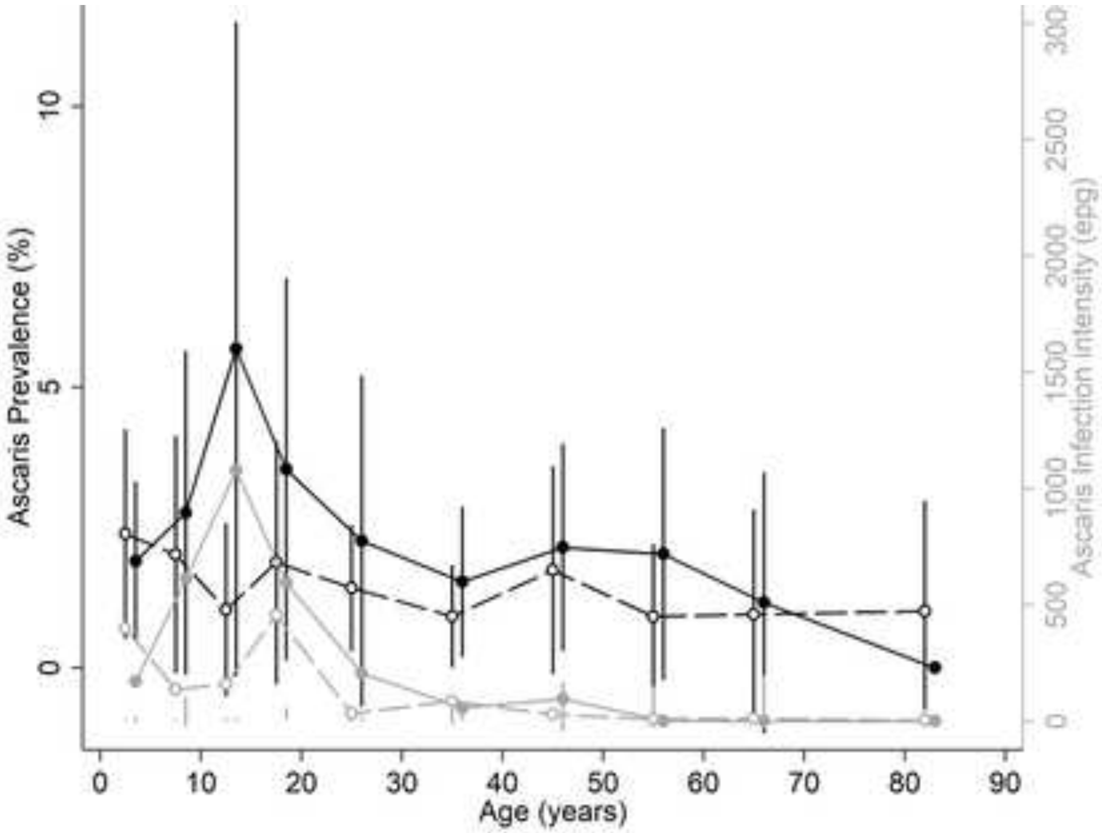

Fig 4. Age-infection profiles for *Ascaris*. (A) Prevalence (black lines) and intensity (grey lines) of *Ascaris* infection by age for males (solid line and circles) and females (dashed lines and empty circles). Vertical bars represent confidence intervals.

Fig 5: Cluster level correlation between prevalence and intensity of hookworm and Ascaris infection in the study population

Click here to access/download;Figure;Fig5.tif

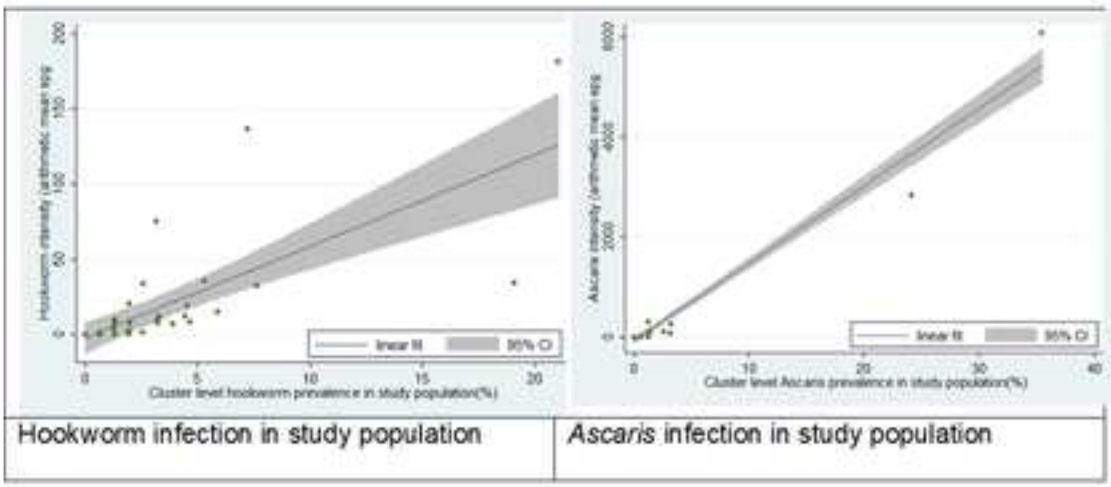

Figure 5: Cluster level correlation between prevalence and intensity of hookworm and *Ascaris* infection in the study population.

Table 1: Comparison of censused population of the DeWorm3 site and longitudinal monitoring cohort (LMC).

	Census<br>n (%) / median (IQR)	LMC<br>n (%) / median (IQR)
~~**Study population (consented)**~~	94,969 (83.99)	6,814 (96.52)
Gender*		
- Female	49,080 (51.68)	3,311 (53.93)
- Male	45,888 (48.32)	2,828 (46.07)
Age distribution*		
- Infants (<1 years)	2,616 (2.75)	-
- Preschool-age children (1-4 years)	11,188 (11.78)	1,184 (19.29)
- School age children (5-14 years)	26,043 (27.42)	1,335 (21.75)
- Adults (15+ years)	54,882 (57.79)	3,620 (58.97)
**Household characteristics**		
Roof materials*		
- Natural materials	5,311 (5.59)	349 (5.68)
- Man-made materials	89,342 (94.07)	5,771 (94.01)
Walls materials		
- Natural materials	22,200 (23.38)	1,359 (22.14)
- Man-made materials	71,258 (75.03)	4,665 (75.99)
Flooring materials		
- Natural materials	16,336 (17.20)	950 (15.47)
- Man-made materials	78,200 (82.34)	5,162 (84.09)
Sources of income<br>-*categories*		
Asset Index quintiles	(n=24,378 households)	(n=6,139 individuals)
Quintile 1 : range [-2.67;-1.84]	5,243 (21.51)	985 (16.04)
Quintile 2 : range [-1.84;-1.19]	4,620 (18.95)	1,043 (16.99)
Quintile 3 : range [-1.19;-0.16]	4,840 (19.85)	1,175 (19.14)
Quintile 4 : range [-0.16; 2.00]	4,884 (20.03)	1,378 (22.45)
Quintile 5 : range [2.00; 12.04]	4,791 (19.65)	1,558 (25.38)
Number of Residents/per Household	5 (4-7)	5 (4-7)
*Missing <5% unless otherwise specified.		

Table 2: Unweighted STH prevalence and intensity of infection by Kato-Katz testing. N=6139

**Kato-Katz Indicator**	**Any STH prevalence (%)**	**Hookworm prevalence (%)**	***Ascaris* prevalence (%)**	***Trichuris* prevalence (%)**
**UNWEIGHTED ESTIMATES**				
**Unweighted Kato-Katz prevalence:** n (%)[1]				
Positive	324 (5.3)	199 (3.2)	126 (2.0)	5 (0.1)
Negative	5,815 (94.7)	5,940 (96.8)	6,013 (98.0)	6,134 (99.9)
**Intensity of infection, among positive Kato-Katz tests: n (%)**[2]				
Light-intensity	258 (79.6)	189 (95.0)	71 (56.3)	4 (80.0)
Moderate-intensity	54 (16.7.)	4 (2.0)	50 (39.7)	0 (0.0)
Heavy-intensity	12 (3.7)	6 (3.0)	5 (4.0)	1 (20.0)
**Unweighted prevalence of moderate/heavy intensity infections: n (%)**				
Moderate- or Heavy-intensity infection	66 (0.2)	10 (0.0)	55 (0.9)	1 (0)

[1] Positivity was defined as the presence of eggs on one of two slides read by laboratory technicians.

[2] Light-intensity infections are defined as 1-4,999 epg of faeces for *Ascaris* infection, 1-999 epg for *Trichuris* and 1-1,999 epg for Hookworms. Moderate-intensity infections are defined as 5,000-49,999 epg for *Ascaris*, 1,000-9,999 epg for *Trichuris* and 2,000-3,999 epg for Hookworms. Heavy-intensity infections are defined as 50,000+ epg for *Ascaris*, 10,000+ epg for *Trichuris* and 4,000+ epg for Hookworms.

Table 3 :Factors associated with the the prevalence and the intensity of hookworm infection in Comé, Bénin : findings from a cross-sectional baseline prevalence survey in the DeWorm3 STH–elimination trial.

**Variables**	**Prevalence**	**Intensity of infection**	**Generalized Logistic Mixed Model Multivariate Analysis †§**		**Negative binomial regression Multivariate Analysis ‡♣**	
	**n (%)**	**median (IQR), [min, max] epg**	**Adjusted Odds Ratio (95%CI)**	**p-value**	**Adjusted Infection Intensity Ratio (95%CI)**	**p-value**
**INDIVIDUAL factors**						
Age	n =6,139	N=6,138		<0.0001		
- Adults (≥15 years)	160/3,620 (4.4)	0(0-0), [0-12,960]	Reference		Reference	
- PreSAC (1-4 years)	27/1,335 (2.0)	0 (0-0), [0-3,552]	0.21 (0.10-0.44)	< 0.001	0.10 (0.03 - 0 .31)	<0.001
- SAC (5-14 years)	12/1,184 (1.0)	0 (0-0), [0-11,100]	0.49 (0.29-0.83)	0.008	0.29 (0.12 – 0.72)	0.01
Gender	n total=6,139	N=6,138		0.004		
- Male	113/2,828 (4.0)	0 (0-0), [0-12,960]	Reference		Reference	
- Female	86/3,311 (2.6)	0 (0-0), [0-9,840]	0.56 (0.38-0.83)	0.004	0.32 (0.16 – 0.64)	0.001
History of deworming during the past year	n total=6,091	N=6,090		0.002		
- No	171/3,687 (4.6)	0 (0-0), [0-12,960]	Reference		Reference	
- Yes	28/2,404 (1.2)	0 (0-0), [0-3,048]	0.45 (0.27-0.75)	< 0.002	0.20 (0.08 – 0.48)	<0.001
Shoe wearing behavior	n =6,091	N=6,090	*		*	
- Shoes	100/3,348 (3.0)	0 (0-0), [0-12,960]				
- No shoes	99/2,743 (3.6)	0 (0-0), [0-11,100]				
Current school attendance	n =6,139	6,138	*		*	
- No	152/4,294 (3.5)	0(0-0), [0-12,960]				
- Yes	47/1,845 (2.5)	0(0-0), [0-11,100]				
**HOUSEHOLD factors**						
Highest education level in the household	n =6,139	6,138	*			
- University/College/Diploma	4/698 (0.6)	0(0-0), [0-228]			Reference	
- No education	93/1,942 (4.8)	0(0-0), [0-12,960]			40.13 (2.47 – 652.77)	0.01
- Primary	50/1,341 (3.7)	0(0-0), [0-11,100]			30.92 (1.86 – 513.88)	0.02

Variables	Prevalence	Intensity of infection	Generalized Logistic Mixed Model Multivariate Analysis †§		Negative binomial regression Multivariate Analysis ‡♣	
	n (%)	median (IQR), [min, max] epg	Adjusted Odds Ratio (95%CI)	p-value	Adjusted Infection Intensity Ratio (95%CI)	p-value
- Secondary	52/2,158 (2.4)	0(0-0), [0-4,764]			19.34 (1.21 – 308.81)	0.04
Quintiles of household asset index	n =6,139	6,138		<0.0001	*	
- 5th quintile (richest)	16/1,558 (1.0)	0(0-0), [0-1,440]	Reference			
- 1st quintile (poorest)	76/985 (7.7)	0(0-0), [0-12,960]	5.03 (2.10-12.01)	<0.001		
- 2nd quintile	51/1,043 (4.9)	0(0-0), [0-4,764]	3.62 (1.51-8.66)	0.001		
- 3rd quintile	38/1,175 (3.2)	0(0-0) [0-9,840]	2.51 (1.05-6.00)	0.02		
- 4th quintile	18/1,378 (1.3)	0(0-0), [0-1,104]	0.94 (0.36-2.51)	0.91		
Head of household's occupation	n =6,139	6,138		0.03		
- Others/ Don't know/Refused	98/4,618 (2.1)	0(0-0), [0-4,764]	Reference		Reference	
- Farmer	89/921 (9.7)	0(0-0), [0-12,960]	1.79 (1.11-2.90)	0.02	3.94 (1.67 – 9.27)	0.002
- Fisher	12/600 (2.0)	0(0-0), [0-516]	0.70 (0.31-1.60)	0.70	0.23 (0.05 - 1.03)	0.06
Observed floor type : natural/manmade	n =6,139	6,138	*			
- Man-made floor material	123/5,162 (2.4)	0(0-0), [0-11,100]			Reference	
- Natural floor material	75/950 (7.9)	0(0-0),[0-12,960]			3.02 (1.36 – 6.70)	0.01
- Other/Don't know/Refused	1/27 (3.7)	0(0-0), [0-48]			3.48 (0.02 - 664.60)	0.65
Urbanization	n =6,134	6,133		0.02		
- Urban	29/2,418 (1.2)	0(0-0), [0-9,840]	Reference		Reference	
- Peri-urban	146/2,922 (5.0)	0(0-0), [0-12,960]	2.59 (1.25-5.40)	0.01	6.18 (1.82 - 20.90)	0.003
- Rural	24/794 (3.0)	0(0-0), [0-3,120]	1.37 (0.58-3.24)	0.48	1.88 (0.42 – 8.46)	0.41
3 tertiles of population density at 1km²	n =6,134	6,133	*		*	
1st tertile [3 ; 542[low	135/2,021 (6.7)	0(0-0) [0-12,960]				
2nd tertile [542 ; 1235[medium	43/2,072 (2.1)	0(0-0), [0-3,624]				
3rd tertile [1235 ; 2528] high	21/2,041 (1.0)	0(0-0), [0-9,840]				
**WASH factors**						
Household water SDG service modified	n =6,135	6,134	*			

Variables	Prevalence	Intensity of infection	Generalized Logistic Mixed Model Multivariate Analysis †§		Negative binomial regression Multivariate Analysis ‡♣	
	n (%)	median (IQR), [min, max] epg	Adjusted Odds Ratio (95%CI)	p-value	Adjusted Infection Intensity Ratio (95%CI)	p-value
- Improved ≤ 30min	141/5,098 (2.8)	0 (0-0),[0-2,124]			Reference	
- Surface water > 30min	0/3 (0.0)	0(0-0), [0-3120]			0	-
- Surface water ≤ 30min	2/16 (12.5)	0(0-0), [0-120]			45.62 (0.76 - 2726.46)	0.07
- Unimproved > 30min	6/53 (11.3)	0(0-0), [0-696]			13.47 (1.62 – 111.55)	0.02
- Unimproved ≤ 30min	39/583 (6.7)	0(0-0), [0-2,124]			1.89 (0.69 - 5.12)	0.21
- Improved > 30 min	11/382 (2.9)	0(0-0), [0-3,120]			0.69 (0.16 – 3.01)	0.62
	n =5,816			0.24		
Household sanitation SDG service	129/2,162 (6.0)	5,815	Reference			
- Open defecation	3/227 (1.3)	0(0-0), [0-12,960]	0.50 (0.13-1.93)	0.32	Reference	
- Unimproved shared	1 /127(0.8)	0(0-0), [0-60]	0.23 (0.02-2.24)	0.20	0.18 (0.01 – 2.26)	0.19
- Unimproved unshared	28/1,666 (1.7)	0(0-0), [0-204]	0.73 (0.38-1.37)	0.33	0.07 (0.002 – 2.16)	0.13
- Improved shared	28/1,634 (1.7)	0(0-0), [50-9,840]	0.48 (0.24-0.98)	0.04	0.44 (0.116 – 1.25)	0.12
- Improved unshared	n = 5,716	0(0-0), [0-1,440]	**	0.46	0.23 (0.07 – 0.70)	0.01
Household Hand washing facility SDG service	64/1,881 (3.4)	5,715	Reference		**	
- No facility	104/3,111 (3.3)	0(0-0), [0-12,960]	0.94 (0.61-1.44)	0.76	Reference	
- Limited	13/724 (1.8)	0(0-0), [0-8,064]	0.61 (0.28-1.34)	0.22	0.83 (0.39 – 1.77)	0.64
- Basic		0(0-0), [0-1,440]			0.44 (0.11 – 1.73)	0.24
**ENVIRONMENTAL Factors**						
Elevation (in meters)	n = 6,134	6,133	**	0.12	*	
1st tertile [-1 ; 15[(low)	63/2,094 (3.0)	0(0-0), [0-12,960]	Reference			
2nd tertile [15 ; 30[(medium)	37/2,300 (1.6)	0(0-0), [0-9,840]	1.07 (0.53-2.17)	0.85		
3rd tertile [30 ; 61] (high)	99/1,740 (5.7)	0(0-0), [0-11,100]	1.73 (0.98-3.06)	0.06		
Proportion of soil that is sand at the surface at 0 cm (%)	n total=6,134	6,133	*		*	
1st tertile [35 ; 55[(low)	37/2,143 (1.7)	0(0-0), [0-1,176]				
2nd tertile [55 ; 64[(medium)	40/2,001 (2.0)	0(0-0), [0-3,624]				

Variables	Prevalence	Intensity of infection	Generalized Logistic Mixed Model Multivariate Analysis †§		Negative binomial regression Multivariate Analysis ‡♣	
	n (%)	median (IQR), [min, max] epg	Adjusted Odds Ratio (95%CI)	p-value	Adjusted Infection Intensity Ratio (95%CI)	p-value
3rd tertile [64 ; 78] (high)	122/1,990 (6.1)	0(0-0), [0-12,960]				
Soil acidity (pH KCL) at everage depth (0-5-15 cm)	n total= 6,134	6,133	*		*	
1st tertile [4.8 ; 5.1[(low)	40/2,810 (2.0)	0(0-0), [0-11,100]				
2nd tertile [5.1 ; 5.2[(medium)	90/1,230 (4.0)	0(0-0), [0-9,840]				
3rd tertile [5.2 ; 5.7] (high)	69/2,094 (3.7)	0(0-0), [0-12,960]				
MODIS daytime land surface temperature mean for 2018 (°celsius)	n = 6,134	6,133	*		*	
1st tertile [26.2 ; 29.6[(low)	71/2,097 (3.4)	0(0-0), [0-11,100]				
2nd tertile [29.6 ; 31.9[(medium)	109/2,407 (4.5)	0(0-0), [0-12,960]				
3rd tertile [31.9 ; 32.8] (high)	19/1,630 (1.2)	0(0-0), [0-3,624]				
MODIS Enhanced Vegetation Index (EVI) mean for 2018	n =6,134	6,133	*		*	
1st tertile [0.04 ; 0.2[(low)	26/2,086 (1.2)	(0-0), [0-9,840]				
2nd tertile [0.2 ; 0.3[(medium)	35/2,061 (1.7)	(0-0), [0-3,624]				
3rd tertile [0.3 ; 0.4] (high)	138/1,987 (6.9)	(0-0), [0-12,960]				
MODIS normalized difference vegetation index (NDVI) mean for 2018	n = 6,134	6,133	**	0.007	**	
1st tertile [0.06 ; 0.3[(low)	27/2,063 (1.3)	(0-0), [0-9,840]	Reference		Reference	
2nd tertile [0.3 ; 0.4[(medium)	33/2,079 (1.6)	(0-0), [0-3,624]	0.72 (0.33-1.56)	0.41	0.44 (0.12 - 1.61)	0.21
3rd tertile [0.4 ; 0.6] (high)	139/1,992 (7.0)	(0-0), [0-12,960]	2.00 (0.93-4.28)	0.07	3.31 (0.85 – 12.93)	0.08
Aridity index	n total=6,134	6,133	*		*	
1st tertile [0.59 ; 0.61[(low)	81/2,083 (3.9)	(0-0), [0-9,840]				
2nd tertile [0.61 ; 0.62[(medium)	50/2,099 (2.4)	(0-0), [0-8,064]				
3rd tertile [0.62 ; 0.65] (high)	68/1,952 (3.5)	(0-0), [0-12,960]				

† Adjusted Generalized logistic mixed model estimating equations with exchangeable correlation structure.
§ 5,366 observations included in fully adjusted model.
‡ Adjusted zero-inflated negative binomial regression model, inflating for sex and age (1–4 years, 5–14 years, 15 years), with an exchangeable correlation matrix.

Variables	Prevalence	Intensity of infection	Generalized Logistic Mixed Model Multivariate Analysis †§		Negative binomial regression Multivariate Analysis ‡♣	
	n (%)	median (IQR), [min, max] epg	Adjusted Odds Ratio (95%CI)	p-value	Adjusted Infection Intensity Ratio (95%CI)	p-value

♣ 5,364 observations included in fully adjusted model.

* Variable dropped from fully adjusted model during model adjustment process using lowest AIC criteria.
** Variable in the final adjusted model but with no significant category

Abbreviation: School Aged Children (SAC), Pre School Aged Children (PSAC), confidence interval (CI), interquartile range (IQR), Moderate Resolution Imaging Spectroradiometer (MODIS)

Click here to access/download;Table;Table4_Final AJFL_EA_26.12.2020.docx

Table 4 : Factors associated with *Ascaris* infection prevalence in Comé, Bénin: findings from a baseline prevalence survey using Kato-Katz technique

Variables	Ascaris Infection n infected (%)	Univariate Analysis Odds Ratio (95% CI)	p-value	Multivariate Analysis Adjusted Odds Ratio (95%CI)	p-value
**INDIVIDUAL factors**					
Age	n = 6,139		0.02		
- Adults (≥15 years)	62/3,620 (1.71)	Reference		Ref	
- PreSAC (1-4 years)	24/1,335 (2.03)	1.3 (0.7-2.2)	0.42	1.6 (0.8-3.1)	0.14
- SAC (5-14 years)	40/1,184 (3.00)	2.0 (1.2-3.3)	0.005	2.0 (1.1-3.6)	0.01
Gender	n = 6,139				
- Male	74/2,828 (2.62)	Reference			
- Female	52/3,311 (1.57)	0.5 (0.3-0.8)	0.003	0.5 (0.3-0.9)	0.02
History of deworming during the past year	n = 6,091			*	
- No	98/3,687 (2.66)	Reference			
- Yes	28/2,404 (1.16)	0.7 (0.4-1.2)	0.24		
Shoe wearing behavior	n = 6,091			*	
- Shoes	53/3,348 (1.58)	Reference			
- No shoes	73/2,743 (2.66)	0.8 (0.5-1.3)	0.41		
Current school attendance	n = 6,139			**	
- No	72/4,294 (1.68)	Reference			
- Yes	54/1,845 (2.93)	2.0 (1.3-3.0)	0.001		
**HOUSEHOLD factors**					
Highest education level in the household	n = 6,139		0.08	**	
- University/College/Diploma	3/698 (0.43)	Reference			
- No education	41/1,942 (2.11)	2.4 (0.6-9.0)	0.21		
- Primary	45/1,341 (3.36)	4.2 (1.1- 16.6)	0.04		
- Secondary	37/2,158 (1.71)	3.1 (0.8-11.9)	0.10		
Quintiles of household asset index	n = 6,139		0.14	**	
- 5th quintile (richest)	13/1,558 (0.83)	Reference			
- 1st quintile (poorest)	43/985 (4.37)	1.8 (0.8- 4.1)	0.15		
- 2nd quintile	30/1,043 (2.88)	1.7 (0.7- 3.9)	0.21		
- 3rd quintile	22/1,175 (1.87)	0.9 (0.4-2.0)	0.72		

Variables	Ascaris Infection n infected (%)	Univariate Analysis Odds Ratio (95% CI)	p-value	Multivariate Analysis Adjusted Odds Ratio (95%CI)	p-value
- 4th quintile	18/1,378 (1.31)	0.9 (0.4-2.3)	0.94		
Head of household's occupation	n = 6,139		0.21	*	
- Others/ Don't know/Refused	62/4,618 (1.34)	Reference			
- Farmer	13/921 (1.41)	0.9 (0.4-1.9)	0.86		
- Fisher	51/600 (8.50)	1.6 (0.9-2.6)	0.10		
Observed floor type : natural/manmade	n = 6,139		0.99	*	
- Man-made floor material	97/5,162 (1.88)	Reference			
- Natural floor material	29/950 (3.05)	1.0 (0.6-1.7)	0.99		
- Other/Don't know/Refused	1/27 (3.7)	-	-		
Urbanization	n = 6,134		0.26	*	
- Urban	69/2,418 (2.85)	Reference			
- Peri-urban	14/2,922 (0.48)	0.4 (0.1-1.2)	0.11		
- Rural	43/794 (5.42)	0.8 (0.5-1.4)	0.51		
3 tertiles of population density at 1km	n = 6,134		0.005	*	
- 1st tertile [3 ; 542[(low)	19/2,021 (0.94)	Reference			
- 2nd tertile [542 ; 1235[(medium)	57/2,072 (2.75)	1.6 (0.8-3.0)	0.14		
- 3rd tertile [1235 ; 2528] (high)	50/2,041 (2.45)	2.9 (1.5-5.9)	0.002		
**WASH factors**					
Household water SDG service modified	n = 6,063		0.97	*	
- Improved ≤ 30min	107/5,098 (2.10)	Reference			
- Surface water > 30min	0/3 (0.0)	1			
- Surface water ≤ 30min	0/16 (0.0)	1			
- Unimproved > 30min	0/53 (0.0)	1			
- Unimproved ≤ 30min	8/583 (1.37)	1.1 (0.4-2.7)	0.85		
- Improved > 30 min	11/382 (2.88)	1.1 (0.5-2.4)	0.84		
-					
Household sanitation SDG service	n = 5,816		0.65	**	
- Open defecation	84/2,162 (3.89)	Reference		Ref	
- Unimproved shared	5/227 (2.20)	1.3 (0.4-4.3)	0.65	1.6 (0.4 - 6.8)	0.51
- Unimproved unshared	2/127 (1.57)	1.2 (0.2-6.9)	0.84	1.1 (0.1 – 11.5)	0.94
- Improved shared	23/1,666 (1.38)	0.9 (0.5-1.7)	0.74	1.1 (0.5 – 2.1)	0.87
- Improved unshared	11/1,634 (0.67)	0.6 (0.3-1.2)	0.16	0.8 (0.3 – 1.8)	0.55

Variables	Ascaris Infection n infected (%)	Univariate Analysis Odds Ratio (95% CI)	p-value	Multivariate Analysis Adjusted Odds Ratio (95%CI)	p-value
Household Hand washing facility SDG service	n = 5,716		0.09	**	
- No facility	13/1,881 (1.80)	Reference		Ref	
- Limited	16/3,111 (0.85)	2.6 (1.2-5.6)	0.01	1.8 (0.8 – 3.6)	0.13
- Basic	71/724 (2.28)	1.9 (0.7-4.8)	0.20	1.3 (0.5 – 3.4)	0.54
**ENVIRONMENTAL Factors**					
Elevation (in meters)	n = 6,134		0.02	*	
1st tertile [-1 ; 15[(low)	88/2,094 (4.20)	Reference			
2nd tertile [15 ; 30[(medium)	25/2,300 (1.09)	0.6 (0.3-1.2)	0.17		
3rd tertile [30 ; 61] (high)	13/1,740 (0.75)	0.3 (0.1-0.7)	0.01		
Proportion of soil that is sand at the surface at 0 cm (%)	n = 6,134		0.09	*	
1st tertile [35 ; 55[(low)	101/2,143(4.71)	Reference			
2nd tertile [55 ; 64[(medium)	9/2,001 (0.45)	0.5 (0.2-1.1)	0.10		
3rd tertile [64 ; 78] (high)	16/1,990 (0.80)	0.5 (0.2-1.1)	0.07		
Soil acidity (pH KCL) at average depth (0-5-15 cm)	n = 6,134		0.001		
1st tertile [4.8 ; 5.1[(low)	14/2,810 (0.69)	Reference		Ref	
2nd tertile [5.1 ; 5.2[(medium)	29/1,230 (1.31)	2.2 (1.0-5.0)	0.06	2.0 (0.9-4.2)	0.20
3rd tertile [5.2 ; 5.7] (high)	83/2,094 (4.42)	4.1 (1.9-8.8)	0.001	4.8 (1.8-13.1)	0.002
MODIS daytime land surface temperature mean for 2018 (°celsius)	n = 6,134		0.001		
1st tertile [26.2 ; 29.6[(low)	115/2,097 (5.48)	Reference			
2nd tertile [29.6 ; 31.9[(medium)	7/2,407 (0.29)	0.1 (0.03-0.4)	0.001	0.12 (0.03-0.44)	0.001
3rd tertile [31.9 ; 32.8] (high)	4/1,630 (0.25)	0.1 (0.02-0.5)	0.005	0.17 (0.03-0.91)	0.038
MODIS Enhanced Vegetation Index (EVI) mean for 2018	n = 6,134		0.36	*	
1st tertile [0.04 ; 0.2[(low)	20/2,086 (0.96)	Reference			
2nd tertile [0.2 ; 0.3[(medium)	67/2,061 (3.25)	1.1 (0.5-2.4)	0.72		
3rd tertile [0.3 ; 0.4] (high)	39/1,987 (1.96)	0.8 (0.3-1.8)	0.54		

Variables	Ascaris Infection<br>n infected (%)	Univariate Analysis<br>Odds Ratio (95% CI)	p-value	Multivariate Analysis<br>Adjusted Odds Ratio (95%CI)	p-value
MODIS normalized difference vegetation index (NDVI) mean for 2018	n = 6,134		0.90	*	
1st tertile [0.06 ; 0.3[(low)	22/2,063 (1.07)	Reference			
2nd tertile [0.3 ; 0.4[(medium)	65/2,079 (3.13)	1.1 (0.5-2.1)	0.86		
3rd tertile [0.4 ; 0.6] (high)	39/1,992 (1.96)	0.9 (0.4-2.0)	0.87		
Aridity index	n = 6,134		0.54	*	
1st tertile [0.59 ; 0.61[(low)	15/2,083 (0.72)	Reference			
2nd tertile [0.61 ; 0.62[(medium)	92/2,099 (4.38)	0.6 (0.2-1.6)	0.33		
3rd tertile [0.65 ; 0.65] (high)	19/1,962 (0.97)	1.0 (0.3-3.3)	0.96		

* Variable dropped from fully adjusted model during model adjustment process using lowest AIC criteria.
** Variable in the final adjusted model but with no significant category

Abbreviation: School Aged Children (SAC), Pre School Aged Children (PSAC), confidence interval (CI), interquartile range (IQR), Moderate Resolution Imaging Spectroradiometer (MODIS)

SUPPLEMENTARY DATA

Table S1: Burden of moderate to high intensity (MHI) STH infection in the study population by age group, during DeWorm3 baseline analysis in Comé, Bénin

Age group	N =6,139	MHI STH	MHI Hookworm	MHI Ascaris	MHI Trichuris
- Adults (≥15 y)	3,620	24 (0.7%)	7 (0.2)	17 (0.5%)	0
- PSAC (1-4 y)	1,335	14 (1.1%)	1 (0.1%)	13 (1.0%)	0
- SAC (5-14 y)	1,184	28 (2.3%)	2 (0.2%)	25 (2.1%)	1 (0.0)

Click here to access/download;Table;Table S2_AJFLkha_EA_20201005_Final AJFL.docx

SUPPLEMENTARY DATA

Table S2: Burden of moderate to high STH infection in infected individuals by age group and gender, during DeWorm3 baseline analysis in Comé, Bénin

	N=66	Hookworm	Ascaris	Trichuris	Total (%)
PSAC	Male	1	5	-	6 (9)
	Female	-	8	-	8 (12.1)
SAC	Male	2	20	1	23 (34.8)
	Female	-	5	-	5 (7.5)
Adults	Male	4	12	-	16 (24.2)
	Female	3	5	-	8 (12.1)
Total		10 (15.1)	55 (83.3)	1(1.5)	66 (100)

 Click here to access/download;Table;Table S3_Final AJFL_EA_26.12.2020.docx

SUPPLEMENTARY DATA

Table S3: Factors univariately associated with hookworm infection in Comé, Bénin: findings from DeWorm3 cluster randomized trial baseline pre-treatment survey using generalized logistic mixed model.

			Factors associated with prevalence of hookworm infection		Factors associated with intensity of hookworm infection	
Variables	Infected by hookworms	Intensity of hookworm infection	Univariate Logistic regression Analysis		Univariate negative binomial analysis	
	n (%)	Median (IQR), [min, max] epg	OR (95% CI)	p-value	IIR (95% CI)	p-value
Individual factors						
Age	n =6,139	n=6,138		<0.001		<0.0001
- Adults (≥15 years)	160/3,620 (4.4)	0(0-0), [0-12,960]	Reference		Reference	
- PSAC (1-4 years)	27/1,335 (2.0)	0 (0-0), [0-3,552]	0.15 (0.07-0.31)	<0.001	0.04 (0.01 -0.13)	<0.001
- SAC (5-14 years)	12/1,184 (1.0)	0 (0-0), [0-11,100]	0.35 (0.20-0.59)	<0.001	0.13 (0.05-0.33)	<0.001
Gender	n total=6,139	n=6,138		0.002		0.0003
- Male	113/2,828 (4.0)	0 (0-0), [0-12,960]	Reference		Reference	
- Female	86/3,311 (2.6)	0 (0-0), [0-9,840]	0.57 (0.40-0.82)	0.002	0.29 (0.15-0.57)	<0.001
History of deworming during the past year	n total=6,091	n=6,090		<0.001		<0.0001
- No	171/3,687 (4.6)	0 (0-0), [0-12,960]	Reference		Reference	
- Yes	28/2,404 (1.2)	0 (0-0), [0-3,048]	0.22 (0.13-0.37)	<0.001	0.05 (0.02-0.12)	<0.001
Shoe wearing behavior	n =6,091	n=6,090		0.85		0.96
- Shoes	100/3,348 (3.0)	0 (0-0), [0-12,960]	Reference		Reference	

- No shoes	99/2,743 (3.6)	0 (0-0), [0-11,100]	1.03 (0.72-1.48)	0.85	0.98 (0.50-1.94)	0.96
Current school attendance	n =6,139	n=6,138		0.29		0.18
- No	152/4,294 (3.5)	0(0-0), [0-12,960]	Reference		Reference	
- Yes	47/1,845 (2.5)	0(0-0), [0-11,100]	0.80 (0.53-1.21)	0.29	0.60 (0.28-1.26)	0.18
Household factors						
Highest education level in the household	n =6,139	n=6,138		0.002		0.0015
- University/College/Diploma	4/698 (0.6)	0(0-0), [0-228]	Reference		Reference	
- No education	93/1,942 (4.8)	0(0-0), [0-12,960]	6.8 (2.21-20.86)	0.001	39.68 (5.64-279.16)	<0.001
- Primary	50/1,341 (3.7)	0(0-0), [0-11,100]	5.0 (1.60-15.60)	0.006	26.57 (3.68-191.56)	0.001
- Secondary	52/2,158 (2.4)	0(0-0), [0-4,764]	3.66 (1.20-11.18)	0.023	15.87 (2.31-108.97)	0.005
Quintiles of household asset index	n =6,139	n=6,138		<0.001		<0.0001
- 5th quintile (high)	16/1,558 (1.0)	0(0-0), [0-1,440]	Reference		Reference	
- 1st quintile (low)	76/985 (7.7)	0(0-0), [0-12,960]	7.14 (3.45-14.78)	<0.001	53.53 (15.18-188.83)	<0.001
- 2nd quintile	51/1,043 (4.9)	0(0-0), [0-4,764]	4.85 (2.38-9.90)	<0.001	22.03 (6.35-76.43)	<0.001
- 3rd quintile	38/1,175 (3.2)	0(0-0) [0-9,840]	2.84 (1.41-5.70)	0.003	7.10 (2.09-24.07)	0.002
- 4th quintile	18/1,378 (1.3)	0(0-0), [0-1,104]	1.19 (0.55-2.54)	0.66	1.32 (0.36-4.80)	0.67
Head of household's occupation	n =6,139	n=6,138		<0.0001		<0.0001
- Others/ Don't know/Refused	98/4,618 (2.1)	0(0-0), [0-4,764]	Reference		Reference	

- Farmer	89/921 (9.7)	0(0-0), [0-12,960]	3.64 (2.21-5.97)	<0.001	18.10 (7.39-44.32)	<0.001
- Fisher	12/600 (2.0)	0(0-0), [0-516]	1.17 (0.54-2.54)	0.69	0.72 (0.17-3.14)	0.67
Observed floor type : natural/manmade	n =6,139	n=6,138		<0.0001		<0.0001
- Man-made floor material	123/5,162 (2.4)	0(0-0), [0-11,100]	Reference		Reference	
- Natural floor material	75/950 (7.9)	0(0-0),[0-12,960]	2.90 (1.86-4.53)	<0.001	11.49 (5.13-25.72)	<0.001
- Other/Don't know/Refused	1/27 (3.7)	0(0-0), [0-48]	2.94 (0.24-35.64)	0.40	4.92 (0.02-1068.60)	0.562
Urbanization	n =6,134	n=6,133		<0.0001		<0.0001
- Urban	29/2,418 (1.2)	0(0-0), [0-9,840]	Reference		Reference	
- Peri-urban	146/2,922 (5.0)	0(0-0), [0-12,960]	4.34 (2.30-8.20)	<0.0001	22.47 (7.15-70.65)	<0.001
- Rural	24/794 (3.0)	0(0-0), [0-3,120]	2.67 (1.09-6.57)	0.03	6.35 (1.23-32.75)	0.03
Population density at 1km	n =6,134	n=6,133		<0.0001		<0.0001
1st tertile [3 ; 542[	135/2,021 (6.7)	0(0-0) [0-12,960]	Reference		Reference	
2nd tertile [542 ; 1235[	43/2,072 (2.1)	0(0-0), [0-3,624]	0.39 (0.23-0.69)	0.001	0.15 (0.05-0.42)	<0.001
3rd tertile [1235 ; 2528]	21/2,041 (1.0)	0(0-0), [0-9,840]	0.15 (0.73-0.32)	< 0.001	0.02 (0.005-0.07)	<0.001
WASH factors						
Household water service	n =6,135	n=6,134		0.001		-
- Improved ≤ 30min	141/5,098 (2.8)	0 (0-0),[0-2,124]	Reference		Reference	
- Surface water > 30min	0/3 (0.0)	0(0-0), [0-3120]	0	-	0	-
- Surface water ≤ 30min	2/16 (12.5)	0(0-0), [0-120]	11.14 (1.20-103.68)	0.034	75.75 (1.31-4390.32	0.04

- Unimproved > 30min	6/53 (11.3)	0(0-0), [0-696]	5.77 (1.58-21.08)	0.008	97.66 (8.79-1085.16)	<0.001
- Unimproved ≤ 30min	39/583 (6.7)	0(0-0), [0-2,124]	2.72 (1.54-4.81)	0.001	10.70 (3.91-29.27)	<0.001
- Improved > 30 min	11/382 (2.9)	0(0-0), [0-3,120]	0.96 (0.43-2.13)	0.914	0.95 (0.22-4.09)	0.95
Household sanitation service	n =5,816	n=5,815		0.0001		<0.0001
- Open defecation	129/2,162 (6.0)	0(0-0), [0-12,960]	Reference	0.044	Reference	
- Unimproved shared	3/227 (1.3)	0(0-0), [0-60]	0.24 (0.06-0.96)	0.064	0.03 (0.002-0.39)	0.01
- Unimproved unshared	1 /127(0.8)	0(0-0), [0-204]	0.12 (0.01-1.13)	<0.001	0.02 (0.0006-0.54)	0.02
- Improved shared	28/1,666 (1.7)	0(0-0), [50-9,840]	0.29 (0.16-0.54)	<0.001	0.08 (0.03-0.23)	<0.001
- Improved unshared	28/1,634 (1.7)	0(0-0), [0-1,440]	0.28 (0.15-0.52)	0.1705	0.07 (0.02-0.20)	<0.001
Household hand washing facility service	n = 5,716	n=5,715				0.007
- No facility	64/1,881 (3.4)	0(0-0), [0-12,960]	Reference		Reference	
- Limited	104/3,111 (3.3)	0(0-0), [0-8,064]	1.18 (0.76-1.83)	0.452	1.30 (0.59-2.86)	0.51
- Basic	13/724 (1.8)	0(0-0), [0-1,440]	0.58 (0.27-1.25)	0.167	0.30 (0.08-1.16)	0.08

Environmental Factors						
Elevation (in meters)	n = 6,134	6,133		0.02		0.003
1st tertile [-1 ; 15[	63/2,094 (3.0)	0(0-0), [0-12,960]	Reference		Reference	
2nd tertile [15 ; 30[	37/2,300 (1.6)	0(0-0), [0-9,840]	0.45 (0.24-0.86)	0.02	0.17 (0.05-0.54)	0.003
3rd tertile [30 ; 61]	99/1,740 (5.7)	0(0-0), [0-11,100]	0.99 (0.54-1.81)	0.97	0.86 (0.28-2.62)	0.79
Soil sand fraction at the surface at 0 cm (%)	n total=6,134	6,133		0.01		0.001

1st tertile [35 ; 55[	37/2,143 (1.7)	0(0-0), [0-1,176]	Reference		Reference	
2nd tertile [55 ; 64[	40/2,001 (2.0)	0(0-0), [0-3,624]	1.14 (0.61-2.12)	0.67	1.50 (0.49-4.61)	0.48
3rd tertile [64 ; 78]	122/1,990 (6.1)	0(0-0), [0-12,960]	2.45 (1.29-4.66)	0.006	8.60 (2.53-29.19)	0.001
Soil acidity at everage depth (0-5-15 cm)	n total= 6,134	6,133		0.0351		0.018
1st tertile [4.8 ; 5.1[	40/2,810 (2.0)	0(0-0), [0-11,100]	Reference		Reference	
2nd tertile [5.1 ; 5.2[	90/1,230 (4.0)	0(0-0), [0-9,840]	1.93 (1.15-3.24)	0.01	3.47 (1.32-9.16)	0.012
3rd tertile [5.2 ; 5.7]	69/2,094 (3.7)	0(0-0), [0-12,960]	1.86 (1.06-3.25)	0.03	4.04 (1.42-11.49)	0.009
MODIS daytime land surface temperature mean for 2018 (°celsius)	n = 6,134	6,133		0.0082		0.0002
1st tertile [26.2 ; 29.6[	71/2,097 (3.4)	0(0-0), [0-11,100]	Reference		Reference	
2nd tertile [29.6 ; 31.9[	109/2,407 (4.5)	0(0-0), [0-12,960]	1.25 (0.72-2.16)	0.43	2.68 (0.97-7.41)	0.057
3rd tertile [31.9 ; 32.8]	19/1,630 (1.2)	0(0-0), [0-3,624]	0.34 (0.14-0.82)	0.02	0.12 (0.02-0.64)	0.013
MODIS Enhanced Vegetation Index (EVI) mean for 2018	n =6,134	6,133		< 0.0001		<0.0001
1st tertile [0.04 ; 0.2[	26/2,086 (1.2)	(0-0), [0-9,840]	Reference		Reference	
2nd tertile [0.2 ; 0.3[	35/2,061 (1.7)	(0-0), [0-3,624]	1.42 (0.74-2.72)	0.29	2.23 (0.70-7.08)	0.174
3rd tertile [0.3 ; 0.4]	138/1,987 (6.9)	(0-0), [0-12,960]	5.22 (2.61-10.45)	< 0.001	42.41 (11.88-151.44)	<0.001
MODIS normalized difference vegetation index (NDVI) mean for 2018	n = 6,134	6,133		< 0.0001		<0.0001
1st tertile [0.06 ; 0.3[	27/2,063 (1.3)	(0-0), [0-9,840]	Reference		Reference	
2nd tertile [0.3 ; 0.4[	33/2,079 (1.6)	(0-0), [0-3,624]	1.20 (0.63-2.29)	0.57	1.56 (0.50-4.88)	0.443

3rd tertile [0.4 ; 0.6]	139/1,992 (7.0)	(0-0), [0-12,960]	4.94 (2.50-9.76)	< 0.001	36.18 (10.25-127.67)	<0.001
Aridity index	n total=6,134	6,133		0.28		0.0977
1st tertile [0.59 ; 0.61[	81/2,083 (3.9)	(0-0), [0-9,840]	Reference		Reference	
2nd tertile [0.61 ; 0.62[	50/2,099 (2.4)	(0-0), [0-8,064]	1.22 (0.61-2.43)	0.57	1.11 (0.30-4.20)	0.872
3rd tertile [0.65 ; 0.65]	68/1,952 (3.5)	(0-0), [0-12,960]	1.84 (0.84-4.00)	0.12	3.64 (0.89-14.83)	0.071

†Generalized estimating equations with exchangeable correlation structure and logit link applied
All data available displayed for "Infected with Hookworm/ total N (%)"
Acronyms: School Aged Children (SAC), Pre School Aged Children (PSAC) , confidence interval (CI), odds ratio (OR), Moderate Resolution Imaging Spectroradiometer (MODIS)

Click here to access/download;Table;Table S4_Final AJFL_EA_26.12.2020.docx

SUPPLEMENTARY DATA

Table S4 : Intra-Class Correlation values

Risk factor analysis	Model	Level of clustering	Intra-Class Correlation (95% IC)*
Hookworm prevalence**	Model with age and sex	Cluster level	0.16 (0.10-0.26)
		Household within Cluster level	0.58 (0.40-0.74)
	Fully adjusted model	Cluster level	0.03 (0.01-0.10)
		Household within Cluster level	0.39 (0.18-0.65)
Ascaris lumbricoides prevalence **	Model with age and sex	Cluster level	0.54 (0.34-0.73)
		Household within Cluster level	0.60 (0.38-0.78)
	Fully adjusted model	Cluster level	0.42 (0.23-0.64)
		Household within Cluster level	0.51 (0.26-0.76)

*The Intraclass correlation statistics reports two intraclass correlations for this three-level nested model. The first is the level-3 intraclass correlation at the cluster level, the correlation between Hookworm or *Ascaris lumbricoides* infection prevalence in the same cluster. The second is the level-2 intraclass correlation at the household-within-cluster level, the correlation between prevalence of infection with *Ascaris lumbricoides* in the same household and cluster.

** Generalized logistic mixed model with exchangeable correlation matrix
