# Supplementary material for: Factors associated with soil-transmitted helminths infection in Benin: Findings from the DeWorm3 study
Source: PLoS Negl Trop Dis. 2021 Aug 17;15(8):e0009646. doi: 10.1371/journal.pntd.0009646 (PMC8396766; doi:10.1371/journal.pntd.0009646)
Supplement: Attachment — Submitted filename: Responses to reviewers_06.05.2021.docx [file pntd.0009646.s008.docx]

**RESPONSES TO THE REVIEWERS**

Reviewer's Responses to Questions

<b>Key Review Criteria Required for Acceptance?</b></br></br>
As you describe the new analyses required for acceptance, please consider the following:</br></br>

<b>Methods</b></br></br>
-Are the objectives of the study clearly articulated with a clear testable hypothesis stated?</br>
-Is the study design appropriate to address the stated objectives?</br>
-Is the population clearly described and appropriate for the hypothesis being tested?</br>
-Is the sample size sufficient to ensure adequate power to address the hypothesis being tested?</br>
-Were correct statistical analysis used to support conclusions?</br>
-Are there concerns about ethical or regulatory requirements being met?</br></br>

Reviewer #1: The methods are mostly very clear. Please see my comments and suggestions in the pdf.

Reviewer #2: - Please provide information about the data sources as supplementary description (not use reference).

Reviewer #3: The methodology of the study is quite appropriate. The following concerns need to be addressed:

1. Why was qPCR not used to assess the stool parasites as PCR has been shown to be more sensitive than microscopy?

**Response:** We preferred Kato Katz because this method was easily adaptable to the field. Diagnosis was done as soon as the samples were collected and transferred to the field lab. Intensity of infection was assessed using eggs count method, converted in eggs per gram (EPG). However, three aliquots per sample have been prepared for subsequent qPCR analysis. To respect the blinding, qPCR will be done after the 3 years of intervention.

2. How was the sample size calculated for this study?

**Response:** The LMC size was selected to provide information to models of STH infection. Benin LMC was assembled using an age- and cluster-stratified random sample of 150 individuals from the census in each cluster (30 PSAC, 30 SAC and 90 adults) for a total of 6,000 individuals (1,200 PSAC, 1,200 SAC and 3,600 adults). *These numbers give us 80% power to detect a prevalence difference of 2.5% between PSAC and SAC and between adults and SAC within each arm, assuming a prevalence of 2% among SAC, alpha=0.05, intracluster correlation of 0.01 and a two-sided test* . This was based on the idea that SAC would have been treated most frequently and the other two groups would have higher pre-intervention prevalence.

3. How was the sampling done - random sampling or any other sampling method?

**Response:** Age- and cluster-stratified random sample of 150 individuals from the census in each cluster (30 PSAC, 30 SAC and 90 adults) for a total of 6,000 individuals (1,200 PSAC, 1,200 SAC and 3,600 adults). Participants were excluded if they reported that they planned to move out of the study area within the study period.

--------------------

<b>Results</b></br></br>
-Does the analysis presented match the analysis plan?</br>
-Are the results clearly and completely presented?</br>
-Are the figures (Tables, Images) of sufficient quality for clarity?</br></br>

Reviewer #1: I believe the analysis does match the analysis plan. The tablets need considerable improvement for clarity. Please see my comments and suggestions in the pdf.

Response: Thank you for your comments and suggestions. We tried to improve the clarity of tables and hope it matches your expectations.

Reviewer #2:

- Please provide the detailed map of research area in this paper not reference.

Response: Thanks for your request. We provided the map in this version of the paper
- Transfer the contents of Descriptive in the Results part to the study design in Materials and Methods part. 
- The interesting point in the table 4 is that Ascaris lumbricoides was more prevalent in fisher (not farmer). Please describe your opinion in the discussion part.

Reviewer #3: The results as described are fine. The study does not examine the association of any hematological parameters with stool parasites including anemia. This is a major drawback.

Response: Thanks for your remark. There is no blood sampling in this study design, so we have no data on anemia.

--------------------

<b>Conclusions</b></br></br>
-Are the conclusions supported by the data presented?</br>
-Are the limitations of analysis clearly described?</br>
-Do the authors discuss how these data can be helpful to advance our understanding of the topic under study?</br>
-Is public health relevance addressed?</br></br>

Reviewer #1: The conclusions are supported by the data and the limitations are well described. The authors do discuss (although mostly in the conclusions) how their findings could help improve the control of STH. Please see my comments and suggestions in the pdf.

Reviewer #2: (No Response)

Reviewer #3: The conclusions are appropriate.

--------------------

<b>Editorial and Data Presentation Modifications?</b></br><br/>
Use  this section for editorial suggestions as well as relatively minor modifications of existing data that would enhance clarity. If the only modifications needed are minor and/or editorial, you may wish to recommend “Minor Revision” or “Accept”.

Reviewer #1: Please see my comments and suggestions in the pdf.

Reviewer #2: - Use the name of any parasites according to the normal journal style (Italic, abbreviation etc.) or uniform style determined by the author in whole manuscript including tables, supplementary data, etc.

Reviewer #3: None.

--------------------

<b>Summary and General Comments</b></br></br>
Use  this section to provide overall comments, discuss strengths/weaknesses of the study, novelty, significance, general execution and scholarship. You may also include additional comments for the author, including concerns about dual publication, research ethics, or publication ethics. If requesting major revision, please articulate the new experiments that are needed.

Reviewer #1: This study confirms several of the risk factors assumed to be related to STH infections. It was very nice to see that so many logical associations were found. The manuscript is, overall, well written but I submit (pdf) some suggestions on how to improve it.

Reviewer #2: (No Response)

Reviewer #3: The study examines the presence of STH and associated socio-economic, environmental and WASH factors. The concerns include lack of any hematological details, lack of examining other risk factors etc.

--------------------

PLOS authors have the option to publish the peer review history of their article (<a href="<https://journals.plos.org/plosntds/s/editorial-and-peer-review-process#loc-peer-review-history>" target="_blank">what does this mean?</a>). If published, this will include your full peer review and any attached files.<br><br>

If you choose “no”, your identity will remain anonymous but your review may still be made public.<br><br>

<b>Do you want your identity to be public for this peer review?</b> For information about this choice, including consent withdrawal, please see our <a href="<https://www.plos.org/privacy-policy>" target="_blank">Privacy Policy</a>.

Reviewer #1: No

Reviewer #2: No

Reviewer #3: No


Figure Files:
While revising your submission, please upload your figure files to the Preflight Analysis and Conversion Engine (PACE) digital diagnostic tool, [https://pacev2.apexcovantage.com](https://pacev2.apexcovantage.com/). PACE helps ensure that figures meet PLOS requirements. To use PACE, you must first register as a user. Then, login and navigate to the UPLOAD tab, where you will find detailed instructions on how to use the tool. If you encounter any issues or have any questions when using PACE, please email us at [figures@plos.org](mailto:figures@plos.org).
 
Data Requirements:
Please note that, as a condition of publication, PLOS' data policy requires that you make available all data used to draw the conclusions outlined in your manuscript. Data must be deposited in an appropriate repository, included within the body of the manuscript, or uploaded as supporting information. This includes all numerical values that were used to generate graphs, histograms etc.. For an example see here: <http://www.plosbiology.org/article/info%3Adoi%2F10.1371%2Fjournal.pbio.1001908#s5>.
 
Reproducibility:
To enhance the reproducibility of your results, PLOS recommends that you deposit laboratory protocols in [protocols.io](http://protocols.io/), where a protocol can be assigned its own identifier (DOI) such that it can be cited independently in the future. For instructions see <http://journals.plos.org/plosntds/s/submission-guidelines#loc-materials-and-methods>

SUMMARY OF REVIEWERS EDITS AND QUESTIONS ON THE PDF FILE

| **N°** | **Original row** | **New row on document with track changes** | **Original sentence** | **Correction suggested by the reviewer** | **Comment/Question of the reviewer** | **Author’s response** |
| --- | --- | --- | --- | --- | --- | --- |
| 1 | 1-2 | 1-2 | Factors associated with soil- transmitted helminth infection in Benin: findings from the DeWorm3 study | Factors associated with soil-transmitted helminths infection in Benin: findings from the DeWorm3 study |  | Corrections made to the manuscript |
| 2 |  |  | The most common STH species of humans include *Ascaris* | The most common STH **infecting** humans **are** *Ascaris* |  | Corrections made to the manuscript |
| 3 | Introduction  35 | 93 | STH are transmitted by eggs present in human feces which in turn contaminate soil | **Soil-transmitted helminths** are transmitted by eggs present in human feces**,** which in turn contaminate soil | Depending on the journal one should avoid starting sentences with abbreviations. Not sure if this applies here but I would write "Soil-transmitted helminths" at the beggining of sentences. Same applies for the rest of the manuscript. | Corrections made to the manuscript |
| 4 | 38-39 | 99-100 | …with the greatest numbers occurring in sub-Saharan Africa, the Americas, China and East Asia. |  | China and East Asia sounds a bit redundant to me. | …with the greatest numbers occurring in sub-Saharan Africa, the Americas, China and **South-East** Asia. |
| 5 | 42-43 | 103-104 | moderate-to-heavy intensity infection **- as** measured by number of eggs per gram of stool **diagnosed** by stool examination. | moderate-to-heavy intensity infection**s**, measured by **the** number of eggs per gram of stool **counted during the** stool examination. |  | Corrections made to the manuscript |
| 6 | 48 | 109 | ….lactating women living in endemic areas, are at highest risk of morbidity due to STH. | …lactating women living in endemic areas, are at highest risk of morbidity due to STH**s**. | Please make sure you use STHs when the sentence reads "soil-transmitted helminths" like this case. | Corrections made to the manuscript |
| 7 | 54-55 | 113 | The WHO Neglected Tropical Disease (NTD) Roadmap and London Declaration have  accelerated **the** progress toward eliminating selected NTDs, **including** lymphatic filariasis  and onchocerciasis, and formalized long-term disease-specific goals for other NTDs. | The WHO Neglected Tropical Disease (NTD) Roadmap and London Declaration have  accelerated progress toward eliminating selected NTDs, **such as** lymphatic filariasis  55 and onchocerciasis, and formalized long-term disease-specific goals for other NTDs. |  | Corrections made to the manuscript |
| 8 | 56 | 115 | Global interest is shifting towards an elimination strategy for other NTDs |  | shifting from what towards elimination? control? | Global interest is shifting **from control** towards an elimination strategy for other NTDs |
| 9 | 60 | 118 | In Benin, all major STH (hookworm sp., *A. lumbricoides* and *T. trichiura*) are a recognized public health problem,…….. | In Benin, all major STHs are a recognized public health problem,…….. | Since you have already listed them above, here you could remove them | Corrections made to the manuscript |
| 10 | 62 | 119-120 | ….results of a recent national mapping exercise…. |  | Perhaps it would be nice to mention the year of this national mapping rather than just saying "recent" | 2013-2015 |
| 11 | 63 | 120 | In Comé District | In **the** Comé District |  | Corrections made to the manuscript |
| 12 | 64 | 121 | school-aged children | SAC |  |  |
| 13 | 66 | 123 | ..despite multiple rounds of school-based MDA with albendazole in 2015 (coverage 59%), 2016 (coverage 78%) and 2017 (coverage 83%).  (13) |  | Multiple rounds of MDA in each year? Or once per year in each year (2015, 2016 and 2017)? This is not entirely clear to me from this sentence | despite **three** rounds of **yearly** school-based MDA with albendazole in 2015 (coverage 59%), 2016 (coverage 78%) and 2017 (coverage 83%). (1) |
| 14 | **Study area and population** 80, 81 | 137 | The study site  selection was based on criteria reported previously. | The study site  selection was based on criteria **that have previously been** reported. |  | Corrections made to the manuscript |
| 15 | 84 | 140 | census of 2012, **with an** estimated yearly growth rate of 2.07%. | census of 2012, **and an** estimated yearly growth rate of 2.**1**%. | For consistency, perhaps use only one decimal point throughout the manuscript so this number would be 2.1% | Corrections made to the manuscript |
| 16 | Study design  94 | 154 | Cluster demarcation |  | By "cluster demarcation" do you mean this division into 40 clusters you describe in the next sentence? This is not 100% clear to me | Yes “cluster demarcation “ is related to the division of the study area into clusters. |
| 17 | 101 & 102 | 160-161 | Adults aged 15 years old  …. 90 adults |  | 15 years to 18 years are not adults so perhaps you can reword this section of the sentence | You are right, participants aged 15 to 18 years old are not adults, at a legal point of view. However in this study we needed to categorize individuals according to those exposed to the standard of care MDA, which is school based. So we have the category of school-aged children (SAC=5-14 years old), then the pre-school aged children (PSAC=1-4 years old) and the adults. Adolescents who are over 14 years old are considered in adults category since like adults they do not have access to standard school based MDA intervention.  We rephrased the sentence like this: “participants aged 15 years old and above (considered as adults in this study),” |
| 18 | 104 | 164 | LMC participants were interviewed |  | The interviews were about the access to WASH, history of deworming etc? If yes, perhaps "LMC participants were interviewed about..." because now it sounds there was an interview AND a survey about WASH etc | LMC participants were interviewed about individual-level STHs risk factors, including a survey of self-reported WASH access and use, history of deworming, and direct observation of WASH facilities and participants’ use of footwear. |
| 19 | 108 | 167 |  |  | How many stool samples? | Individuals participating in the LMC agreed to provide one stool sample for immediate analysis using the Kato-Katz method |
| 20 | 109 | 118 | ……….using the Kato-Katz method (16) annually for the duration of the study. | ……….using the Kato-Katz method. (16) |  | Corrections made to the manuscript |
| 21 | 116 | 175 | reported in eggs per gram |  | You mean technicians recorded egg counts during the Kato-Katz, which were later converted into EPG?  I just noticed that right after the formulas below you define EPG. Maybe here it's enough to say that lab technicians recorded egg counts for each parasite seperately. | Two slides were prepared from each sample, and each slide was examined by two experienced lab technicians who recorded egg counts for each parasite separately. |
| 22 | 118 | 190-197 | A subset of 10% of slides was randomly selected for quality assurance by a laboratory  manager. |  | How did you do the quality control? Were technicians blinded to the slide ID? Also, how did you do the quality control for hookworm? Was it all done within 60min before the eggs cleared? | Additional information is provided regarding Kato Katz quality assurance process. A summary table is provided in supplementary data (S1 Table).  No, the technicians were not blinded to the slide ID. However identification was not done manually , but using a QR code who was flashed with a smartphone .  About quality control for hookworm, your concern about the possible clearance of the eggs is legitimate. Sensitivity of Kato-Katz can decrease by almost 50% for detection of hookworm when refrigeration is delayed by more than 3h. A delay of 1 day from refrigeration to laboratory processing can also reduce the sensitivity for hookworm by 50%.(2)  In this regard, the DeWorm3 field workers were intensively sensitized about this matter, in order to shorten this delay as much as possible.  Sentence rephrased:  After stool samples collected from enrolled LMC participants were tested by Kato-Katz (KK), they were aliquoted into three samples for storage and eventual quantitative polymerase chain reaction (qPCR) testing. Quality assurance (QA) was conducted to ensure data quality of KK testing. A subset of 10% of slides was randomly selected for reading by the laboratory supervisor and compared against the original readings (Table S5). O Slides were chosen randomly for checking via the SurveyCTO software. All slides reported as positive were cross-checked by the supervisor to confirm the STH species reported by each reader. Routine checks of a selection of slides reported as negative were also carried out by the supervisor for verification. |
| 23 | 129-130 | 185-186 | In cases of co-infection, the sample was counted to calculate the cumulative  prevalence, and prevalence and intensity assessed separately for each species. |  | This sentence is not clear, please reformulate | In cases of co-infection, prevalence and intensity were assessed separately for each species. The sample was counted to calculate the cumulative prevalence. |
| 24 | 136-137 | 200-201 | The primary outcomes were individual-level infection status for each STH type (positive / negative) and intensity of infection in eggs per gram (epg). | The primary outcomes were individual-level infection status for each STH type (positive / negative) and intensity of infection. | Usually one has only one primary outcome... but if you have defined this like this in the protocol I guess then you would need to keep it like this. | Corrections made to the manuscript |
|  | 143-144 | 208-210 | …environmental factors (elevation, soil  sand fraction, soil acidity at average depth (0-5-15 cm), MODIS…) |  | [ <- because you are using parenthesis within parenthesis perhaps you should use the straight ones like you did below "(cding [residents/room], WASH variables included in the ..."  The same applied to the other cases in this sentence and throughout the manuscript | Corrections made to the manuscript |
| 25 | 145 | 160 | MODIS Enhanced Vegetation Index |  | What is MODIS short for? | Moderate Resolution Imaging Spectroradiometer  MODIS (or Moderate Resolution Imaging Spectroradiometer) is a key instrument aboard the [Terra](http://terra.nasa.gov/) (originally known as EOS AM-1) and [Aqua](http://aqua.nasa.gov/) (originally known as EOS PM-1) satellites. Terra's orbit around the Earth is timed so that it passes from north to south across the equator in the morning, while Aqua passes south to north over the equator in the afternoon. Terra MODIS and Aqua MODIS are viewing the entire Earth's surface every 1 to 2 days, acquiring data in 36 spectral bands, or groups of wavelengths (see MODIS Technical Specifications). These data will improve our understanding of global dynamics and processes occurring on the land, in the oceans, and in the lower atmosphere. MODIS is playing a vital role in the development of validated, global, interactive Earth system models able to predict global change accurately enough to assist policy makers in making sound decisions concerning the protection of our environment. |
| 26 | 155 | 217 | Distance to the closest water source and sharing status for sanitation were also collected. |  | Distance between what and the closest water source? Distance from the household? | Yes , distance from the household |
| 27 | 223 | 279 | ….any adults (≥15 years of age) |  | Same comment: 15-17 year olds are not adults.. | Question previously answered |
| 28 | 224 | 279 | …presenting moderate to heavy intensity STH infection.. |  | Or "moderate-to-heavy" as above? | Yes, “moderate-to-heavy". Corrections made to the manuscript |
| 29 | 226 | 281 | …were treated with albendazole by study staff….. |  | So "adults" with light infections did not receive treatment? If yes, why? | Adults with light infections did not receive treatment with albendazole for several reasons:   (i) In the control clusters, only SAC should be treated with albendazole. (ii) These light intensity infections do not represent any threat for adults health |
| 30 | 233 | 287 | Characteristics of the longitudinal monitoring cohort (LMC) population | Characteristics of the LMC population |  | Corrections made to the manuscript |
| 31 | 234 | 289 | ….censused population of the DeWorm3 site are presented in Table 1, and Fig 1 presents the study flow chart. | …censused population of the DeWorm3 site are presented in Table 1. Fig 2 presents the study flow chart. |  | Corrections made to the manuscript |
| 32 | 236-238 | 290-291 | In those households 7,045 individuals were present, among whom 6,814 consented to participate in the LMC  cohort. |  | So only about 1 person per household was selected? Or there were households where more than one person was selected? | In most of households only 1 person was selected. However there were households where more than one person was selected.  Median=1 IQR=1-2  Range=1-5 |
| 33 | 240 | 294 | sample could not be collected after 3 visits | sample could not be collected after **three** visits |  | Corrections made to the manuscript |
| 34 | 244 | 297 | In total 6,139 tests had two slides read by laboratory technicians. |  | tests or samples? | Tests. |
| 35 | 244 | 297 | In total 6,139 tests had two slides read by laboratory technicians. |  | Before you used the term "lab technicians". Just make sure you use one consistently | Corrections made to the manuscript |
| 36 | 298-299 |  | A random subset of Kato-Katz tests was selected for reading by the supervisor and compared against the original readings for quality assurance |  | This random set is the 10%, correct? Perhaps mention that here and, if possible, provide more detail on how this was done: what discrepancies were considered acceptable and which ones were not? What did you do when there was an unacceptable discrepancy? | For each sample two KK slides were made and read by a single reader. The controllers were responsible for quality assurance aspects. Slides were chosen randomly for checking via the SurveyCTO software. All slides reported as positive were cross-checked by the supervisor to confirm the STH species reported by each reader. Routine checks of a selection of slides reported as negative were also carried out by the supervisor for verification.  Agreement between original Kato-Katz reading and QA reading was 99.3% |
| 37 | 249 | 316 | Among the 6139 individuals tested by Kato-Katz, | Among the **6,139** individuals tested by Kato-Katz, |  | Corrections made to the manuscript |
| 38 | 250 | 269 | due to hookworm spp | due to hookworm | I think you can remove the spp. as you have already mentioned above it's two species and, most often, people just write "hookworm" | Corrections made to the manuscript |
| 39 | 251 | 318 | *Trichuris* was found in just 5 (0.1%) individuals | *Trichuris* was **only** detected in **five** (0.1%) individuals |  | Corrections made to the manuscript |
| 40 | 252 | 319 | Six individuals were co-infected with hookworm and *Ascaris*. | Six individuals were co-infected with hookworm and *Ascaris*. **lumbricoides** | (Please make sure that throughout the manuscript you use the full species names.) | Corrections made to the manuscript |
| 41 | 252 | 319 | Due to the small number of *Trichuris* infections | Due to the small number of *Trichuris. trichiura* infections |  | Corrections made to the manuscript |
| 42 | 253 | 320 | …only analyses focused on hookworm spp. and *Ascaris* are presented. | only **the** analyses focused on hookworm and *Ascaris* are presented. |  | Corrections made to the manuscript |
| 43 | 254 | 321 | ….54 (16.7%) moderate-intensity | ….54 (16.7%) **were** moderate-intensity |  | Corrections made to the manuscript |
| 44 | 255 | 322 | …(3.7%) heavy intensity infections | …(3.7%) **were** heavy intensity infections |  | Corrections made to the manuscript |
| 45 | 256 | 322-323 | Hookworm spp. were more prevalent in adults | Hookworm **was significantly** more prevalent in adults |  | Corrections made to the manuscript |
| 46 | 257 | 324 | SAC were more frequently infected with *Ascaris* compared to PSAC or adults | SAC were  **significantly** more infected with *Ascaris* compared to PSAC or adults |  | Corrections made to the manuscript |
| 47 | 257 | 325 | (3% *versus* 2%  *versus* 1.7% respectively, Chi2, p=0.02) | (3**.0**% *versus* 2.**0**%  *versus* 1.7% respectively, Chi2, p=0.02) | .0  (Correct? Please make sure you use the same decimal points everywhere) | Corrections made to the manuscript |
| 48 | 259 | 326 | ….females was infected with hookworm spp. | ….females was infected with hookworm. |  | Corrections made to the manuscript |
| 49 | 262 | 344 | The median egg density for hookworm spp. was 108 eggs per gram (epg) |  | You have already mentioned EPG several times before so there is no need to redefine the abbreviation - please make sure that you only define it once. Also, I believe "EPG" in capital letters is the most common form so perhaps you could change to that? The most important, however, is to be consistent in the entire paper | Corrections made to the manuscript |
| 50 | 263 | 344-352 |  |  | In my opinion, there are too many numbers in this paragraph. I suggest you highlight the most important numbers and just refer to the Tables (I believe you do not refer to any table in this paragraph...) | To decrease the numbers in the text, we removed the ranges from the results. Only median (IQR) are presented. |
| 51 | 273 | 353 | Moderate to heavy intensity (MHI) infections |  | Again "moderate-to-heavy" or "Moderate to heavy" - one needs to be consistent. Please double check this throughout the text.  Is this the first time you define MHI? | Moderate-to-heavy |
| 52 | 273 | 296 | Moderate to heavy intensity (MHI) infections were found in 66/6,139 individuals overall |  | 66 out of 6?? | No. 66 out of 6,139. |
| 53 | 274-275 | 354-356 | amongst whom 10 (0.2%) MHI with hookworm spp., 55 (0.9%) MHI with *Ascaris* and 1 (<0.1%) MHI with *Trichuris* (Table 2). | amongst whom 10 (0.2%) **had** MHI with hookworm, 55 (0.9%) **had** MHI with *Ascaris* and 1 (<0.1%) **had** MHI with *Trichuris trichiura*(Table 2). |  | Corrections made to the manuscript |
| 54 | 276 | 356 | with 2.1% (25/1,184) prevalence of MHI of *Ascaris* |  | 25 out of 1? | No, 25 out of 1,184 |
| 55 | 287 | 367-368 | The prevalence was similar in males and females among PSAC and SAC, but in adults, the prevalence in males was higher | The prevalence was similar in males and females among PSAC and SAC. **However**, in adults the prevalence in males was higher |  | Corrections made to the manuscript |
| 56 | 294 | 375 | Intensity of *Ascaris* | **The i**ntensity of *Ascaris* |  | Corrections made to the manuscript |
| 57 | 301-302 | 389-390 | This correlation was strong for both hookworm spp. (ρ=0.73, p<0.0001) and *Ascaris* (ρ=0.98, p<0.0001). |  | Shouldn't it be a normal "p" like in the other paragraphs?  typically it's enough to say p<0.01 | Here we are talking about the Pearson correlation coefficient. So we change the «ρ » (Rho) –Spearman coefficient into « r » -Pearson coefficient. |
| 58 | 306-307 | 397-398 | Here, the results of multivariable analyses are presented (Table 3). | **In this section, we present** the results of multivariable analyses (Table 3). |  | Corrections made to the manucript |
| 59 | 308 | 399 | At the individual level… | At **an** individual level |  | Corrections made to the manuscript |
| 60 | 309 | 400 | Hookworm spp. | hookworm |  | Corrections made to the manuscript |
| 61 | 324 | 413 | Among WASH factors, household sanitation, and especially open defecation, was found to be strongly associated with hookworm infection. | Among WASH factors, household sanitation, and open defecation **were** found to be strongly associated with hookworm infection. |  | Corrections made to the manuscript |
| 62 | 330 | 416 |  |  | Make sure to refer to the tables and supplementary files - I believe you are missing some references in these different section of the results |  |
| 63 | 332 | 434 | ….95%CI 0.12 – 0.72, p=0.01 | ….95%CI 0.12 – 0.72, p=0.01**)** |  | Corrections made to the manuscript |
| 64 | 335 | 436 |  |  | Please remove these spaces between paragraphs | Corrections made to the manuscript |
| 65 | 373 | 483 | Comparison of the Intra-Class Correlation values | Comparison of the Intra-Class Correlation **(ICC)** values |  | Corrections made to the manuscript |
| 66 | 387 | 496 | …… *Ascaris* infection prevalence respectively, in the fully adjusted model.  . | ……. *Ascaris lumbricoides* infection prevalence**,** respectively. |  | Corrections made to the manuscript |
| 67 | 388 | 497 | ..fully adjusted multivariable final model, |  | Is this defined in the methods? | In the methods we talked about fully adjusted model. Sentence corrected in the results to match methods :  …fully adjusted model. |
| 68 | 406 | 513-514 | …the intensity of hookworm infection was also decreased.. | …the intensity of hookworm infection also decreased.. |  | Corrections made to the manuscript |
| 69 | 407 | 514 | … was available at less than 30 minutes distance. | … was available at less than 30 minutes **walking** distance |  | Corrections made to the manuscript |
| 70 | 411 | 517 | …and soil  transmitted helminths. | and **STH**. |  | Corrections made to the manuscript |
| 71 | 411 | 518-519 | …the ENVISION program |  | Define this and USAID | USAID defined in the manuscript.  Cannot Define ENVISION |
| 72 | 413-414 | 520-521 | A nationwide STH prevalence survey was completed in 2015, that reported 20% prevalence (13) in school-aged children in Comé district. | A nationwide STH prevalence survey **that** was completed in 2015, reported 20% prevalence (13) in **SAC** in **the** Comé District |  | Corrections made to the manuscript |
| 73 | 415 | 521 | …3 rounds of school-based MDA with albendazole | **…three** rounds of school-based MDA with albendazole |  | Corrections made to the manuscript |
| 74 | 417 | 523 | …primarily targeting school-age (SAC) and pre-school age children (PSAC) | … primarily targeting SAC and PSAC |  | Corrections made to the manuscript |
| 75 | 420 | 526 | …Mebendazole | **…m**ebendazole |  | Corrections made to the manuscript |
| 76 | 421 | 527 | …starting from the 2nd trimester | starting from the **second** trimester |  | Corrections made to the manuscript |
| 77 | 422 | 527 | In the context described, | In th**is** context, |  | Corrections made to the manuscript |
| 78 | 425 | 530 | …as prescribed by the WHO Neglected Tropical Disease (NTD) Roadmap | …as prescribed by the WHO NTD Roadmap |  | Corrections made to the manuscript |
| 79 | 428-429 | 533 | (5.2% versus 20.0% respectively, p<0.001). |  | add the reference here | Reference added. (13)  Ibikounlé M, Onzo-Aboki A, Doritchamou J, Tougoué J-J, Boko PM, Savassi BS, et al. Results of the first mapping of soil-transmitted helminths in Benin: Evidence of countrywide hookworm predominance. Aksoy S, editor. PLoS Negl Trop Dis. 2018 Mar 1;12(3):e0006241. |
| 80 | 434 | 539 | …compared to the findings of the national STH mapping 3 years earlier. | … compared to the findings of the national STH mapping **three** years earlier. |  | Corrections made to the manuscript |
| 81 | 436 | 540-541 | … instead of in schools, | instead of schools, |  | Corrections made to the manuscript |
| 82 | 440 | 544 | …other STH species in SAC could also be that STH rate of reinfection | …other STH species in SAC could also be that **the** STH rate of reinfection |  | Corrections made to the manuscript |
| 83 | 441 | 545 | with a faster reinfection with *Ascaris* than hookworm spp. |  | reference? | The reference [26] is cited in the next sentence.  Jia T-W, Melville S, Utzinger J, King CH, Zhou X-N. Soil-transmitted helminth reinfection after drug treatment: a systematic review and meta-analysis. PLoS Negl Trop Dis. 2012 Jan 1;6(5):e1621. |
| 84 | 443-445 | 547-548 |  |  | for a discussion, adding CIs of other studies is a bit too much... | Corrections made to the manuscript (CIs removed) |
| 85 | 450 | 553 | hookworms harbored by adults ranged from 70 to 85%, | hookworms harbored by adults range**s** from 70 to 85%, |  | Corrections made to the manuscript |
| 86 | 451 | 553 | ..study in Indonesia show | study in Indonesia show**s** |  |  |
| 87 | From 455 to 468 | 557-570 |  |  | This belongs in the introduction | This paragraph is to explain according to our opinion and using references, why Hookworm prevalence was higher in adults while *Ascaris lumbricoides* prevalence was higher in children. |
| 88 | 457 | 559 | The three species of STH (*A. lumbricoides*, *T. trichiura*, hookworm spp.) have relatively similar cycles… | The three species of STH have relatively similar cycles… |  | Corrections made to the manuscript |
| 89 | 458 | 560 | relatively similar cycles involving the presence of adult worms in the intestine, however the main mode of transmission of *Ascaris* and… | …relatively similar cycles involving the presence of adult worms in the intestine. **H**owever**,** the main mode of transmission of *Ascaris* and… |  | Corrections made to the manuscript |
| 90 | 492 | 591 | …quality of water seems to affect intensity of hookworm infection. | …**the** quality of water seems to affect **the** intensity of hookworm infection**s**. |  | Corrections made to the manuscript |
| 91 | 505 | 604 | This study had a number of strengths, including the large population size, completeness and quality of data and the high level of quality control for Kato-Katz diagnosis, with double reading by the lab technicians of the whole sample with an additional control of a subset of samples by a senior skilled parasitologist. |  | This first sentence could be cut into two, it's too long as is | This study had a number of strengths, including the large population size, completeness and quality of data and the high level of quality control for Kato-Katz diagnosis. Actually Kato Katz’s QC consisted in double reading by the lab technicians of the whole sample with an additional control of a subset of samples by a senior skilled parasitologist. |
| 92 | 525-526 | 622-623 | Improved unshared sanitation and access to improved water sources are associated with lower prevalence and/or intensity of hookworm infection. |  | This reads like a result - try to make it more into action, "WASH should be improved because we found that..." | Correction made to the manuscript |
| 93 | 530 | 628 | …a-year for three years in this setting and combined with these results will inform… | …a-year for three years in this setting**s**. **W**ith these results **we** will inform… |  | Corrections made to the manuscript |
| 94 | Figure 2 | Figure 3 |  |  | I would have only 5 horizontal lines for the different prevalence %s: 1, 2, 3, 4, and 5% without the decimal point. | Corrections made to the graph |
| 95 | Figure 4 | Figure 5 |  |  | Please add the full name of the parasite species and make it italic. Otherwise, this is a nice figure! | Corrections made to the graph |
| 96 | Figure 5 | Figure 6 |  |  | The axis labels need some fixing: missing a parenthesis, missing a space, full species name and italic | Corrections mage to the graph |
| 97 | Table 1 |  |  |  | Center the text in the columns, now it's partly centered and partly to the left | Text centered |
| 98 | Table 1 |  | **Study population (consented)** | Participants | This is a bit confusing because you say "Study population (consented)" but only those in the LMC column are the study population right? | Table revised for more clarity. |
| 99 | Table 1 |  | Census  94,969 (83.99)  LMC  6,814 (96.52) | Census  94,969 (84.0)  LMC  6,814 (96.5) | In my opinion 1 decimal point (ie 84.0 and 96.5) is plenty. Two decimal points makes the table a bit too cded and doesn't add much information | Correction made to the table |
| 100 | Table 1 |  | Adults (15+ years) |  | same comment as before | Answer provided (item 17) |
| 101 | Table 1 |  | Quintile 1 : range [-2.67;-1.84] |  | Not clear what these numbers are, please specifiy somewhere or remove form the table and present it in the results section. | These numbers represent ranges of the quintiles of the asset index, a wealth score constructed using Demographic and Health Survey methods. The lowest quintiles represent poor wealth asset (poorest) and the highest represent good wealth asset (richest). |
| 102 | Table 1 |  | Number of Residents/per Household |  | Maybe you can just have this in the text (results section) because it's the only thing forcing you to have a strange column header "n (%) /  median (IQR)" which can be a bit confusing | As the purpose of this table is to compare the distribution of LMC study population to the censused population it is useful to see these two values side-by-side in a table. |
| 103 | Table 2 |  | **Any STH**  **prevalence**  **(%)** |  | Do you really mean to have "prevalence (%)" here? Even if you have then "**Unweighted Kato-Katz prevalence: n (%)1**" below... Or you just want to have the "any parasite" and then each parasite separately in each column? | You are right.  Any STH, then hookworm, Ascaris lumbricoides and Trichuris trichiura. Correction made to the manuscript. |
| 104 | Table 2 |  | **Unweighted Kato-Katz prevalence: n (%)** |  | This is also a bit confusing for me because the prevalence is what is in the brackets right? Maybe you mean "Number of participants (prevalence)"?  The same applies to the following two titles in this table |  |
| 105 | Table 2 |  | 1 Positivity was defined as the presence of eggs on one of two slides read by laboratory  technicians. |  | I would remove these notes from the table and add them to a "Notes:" section right below, free text not in the table. | Note section created, separated from the table. |
| 106 | Table 2 |  | Heavy-intensity infections are defined as 50,000+ epg for *Ascaris* |  | I would prefer ">50,000 EPG" rather than "50,000+ epg" | Done |
| 107 | Table 3 |  | **Table 3** :Factors associated with the the prevalence and the intensity of hookworm infection in Comé, Bénin : findings from a cross-sectional  baseline prevalence survey in the DeWorm3 STH–elimination trial. |  | Overall this table is not very clear to me... Please try to improve its clarity | Changes made to the table to improve its clarity |
| 108 | Table 3 |  | Prevalence n(%) |  | Again, if I understoof correctly, the prevalence is the % you mention already below so I would not call this column "prevalence" | Number of participants with hookworm infection (prevalence [%]) |
| 109 | Table 3 |  | Intensity of infection  **median (IQR), [min,**  **max] epg** |  | Intensity of infection or EPG? | Intensity of hookworm infection (EPG) |
| 110 | Table 3 |  | **Adjusted**  **Infection Intensity**  **Ratio (95%CI)** |  | Define CI somewhere in the table or its legend | CI defines in the notes |
| 111 | Table 4 |  | **Table 4 :** Factors associated with *Ascaris* infection prevalence in Comé, Bénin: findings from a baseline prevalence survey using Kato-Katz  technique |  | Same comment as for Table 3 | **Number of participants with *Ascaris lumbricoides* infection (prevalence [%])** |
| 112 | Table 4 |  | Ascaris Infection  n infected (%) |  | Infection infected? | **Number of participants with *Ascaris lumbricoides* infection (prevalence [%])** |
| 113 | Table S2 |  | **Table S2**: Burden of moderate to high STH infection in infected individuals by age group and  gender, during DeWorm3 baseline analysis in Comé, Bénin |  | Could this table be combined with Table S1?  Why is the N so different from Table S1? It is not very clear what the difference is between the two populations | Table S1 is now Table S2: **Table S2:** Burden of moderate-to-heavy intensity (MHI) STH infection in the study population by age group, during DeWorm3 baseline analysis in Comé, Bénin  Table S2 is now S3: **Table S3**: Burden of moderate-to-heavy intensity STH infection among infected individuals by age group and gender, during DeWorm3 baseline analysis in Comé, Bénin  Difference between the two populations: Table S2 is related to MHI in the whole study population, while table S3 is related to MHI among only infected participants (positive to Kato-Katz). |
| 114 | Table S3 |  | Infected by  hookworms |  | Either "Hookworm" or "Hookworms" - please be consistent |  |
| 115 | Table S4 |  | Ascaris lumbricoides  prevalence ** |  | Make sure all the scientific names are in italic |  |
| 116 | Table S4 |  | *The Intraclass correlation statistics reports two intraclass correlations for this three-level  nested model…………. |  | I would create separate place for notes, and not have them in the table |  |

**References**

1.	Ibikounlé M, Onzo-Aboki A, Doritchamou J, Tougoué J-J, Boko PM, Savassi BS, et al. Results of the first mapping of soil-transmitted helminths in Benin: Evidence of countrywide hookworm predominance. Aksoy S, editor. PLoS Negl Trop Dis. 2018 Mar 1;12(3):e0006241.

2.	Dacombe RJ, Crampin AC, Floyd S, Randall A, Ndhlovu R, Bickle Q, et al. Time delays between patient and laboratory selectively affect accuracy of helminth diagnosis. Trans R Soc Trop Med Hyg. 2007 Feb;101(2):140–5.
