# Supplementary material for: Factors associated with soil-transmitted helminths infection in Benin: Findings from the DeWorm3 study
Source: PLoS Negl Trop Dis. 2021 Aug 17;15(8):e0009646. doi: 10.1371/journal.pntd.0009646 (PMC8396766; doi:10.1371/journal.pntd.0009646)
Supplement: Attachment — Submitted filename: Responses to reviewers_30.06.2021.docx [file pntd.0009646.s009.docx]

ANSWERS TO THE REVIEWER’S QUESTIONS

Reviewer's Responses to Questions

**Key Review Criteria Required for Acceptance?**

As you describe the new analyses required for acceptance, please consider the following:

**Methods**

-Are the objectives of the study clearly articulated with a clear testable hypothesis stated?

-Is the study design appropriate to address the stated objectives?

-Is the population clearly described and appropriate for the hypothesis being tested?

-Is the sample size sufficient to ensure adequate power to address the hypothesis being tested?

-Were correct statistical analysis used to support conclusions?

-Are there concerns about ethical or regulatory requirements being met?

Reviewer #4:

-The objectives of the study are clearly articulated

-Since the study is part of a longitudinal study and that the current report was only on the baseline data, the design appropriate should be focus on a cross sectional design, irrespective of the main study. So this should be specified or clarified

**Author response:** We thank the reviewer for this important notice.

We clarified this in the study design: “We are reporting in this paper the findings of the baseline cross-sectional survey, the first of the five annual surveys that will we done as part of the LMC.”

-The study population need more details and not just a reference to a published manuscript. Particularly activities carried out by the population which might be a risk factor for STH infection, as well as the Socio-economic level of the study population.

**Author** **response**:

We thank the reviewer for this remark. We added information about the study population in the current version of the manuscript.

The population of Comé is mostly rural, with agriculture and fishing as the main activities. The agricultural, breeding, market gardening and fish farming areas cover 73% of the territory. The commune of Comé is crossed by a dense hydrographic network with 22% of floodable zones.

The socio-economic level of the study population has been explored and an asset index. This indicator describing level of wealth was computed using a Principal Component Analysis method. Several variables describing ownership of goods were used (electricity, generator, radio, stove, television, computer refrigerator, sofa, mattress, solar lamp, ceiling fan, watch, mobile phone, bicycle, motorcycle, rickshaw, cart, car, walls type, cows, cattle, horses, goats, sheep, pigs, chicken, poultry) and the continuous asset index obtained was categorized in five quintiles. The description of the study population according to their asset index is shown in the results.

-The sample size is robust enough .

**Author response:** We thank the reviewer for this comment.

-The author chose adult group >15 years old, can they give a rational underline this choice? Indeed In the same document 14-18 years old are considered as adolescent,. This can confuse a reader, if rational was not provided.

**Author response**:

Dear reviewer, we thank you for this remark.

Participants aged 15 to 18 years old are not adults, from a legal point of view.

The rationale behind the choice of the PSAC, SAC and adults groups in the following: as Deworm3 trial is designed to help improve WHO policies, we needed to categorize individuals according to those exposed to the standard of care MDA against STH, which is school based. So we have the category of the pre-school aged children (PSAC=1-4 years old), the school-aged children (SAC=5-14 years old), then participants who are over 14 years old including adolescents and adults. Adolescents are considered in adults category since like adults they do not have access to standard school based MDA intervention.

We rephrased the sentence like this: “participants aged 15 years old and above (considered as adults in this study)”……

-Only Kato-katz technique was performed in this study, while for hookworm, more sensitive stool culture tests are available and recommended including McMaster technique, or Harada-Mori which provide more comprehensive results. We can understand that in a routine RT-PCR may not be used, but umblinding reason provided by the author should be applied to a microscopy as well. Please provide more information on the study limitations regarding the use of the kato-katz only in a discussion section.

**Author response**:

Dear reviewer, many thanks for your contribution about limitations of Kato-Katz technique.

The Kato-Katz technique has been used in this survey due to the huge number of samples (6,000) collected to be analyzed in a short time. It is the most broadly used technique in STH epidemiological surveys and recommended by WHO for diagnosing and quantifying soil-transmitted helminth (STH) – as it provides a standardized reading (eggs per gram of faeces (epg)) and the technique can be taught to laboratory microscopists relatively easily. However the sensitivity of the technique is low, leading to false negative results in low endemicity settings. To address this concern, we did a double reading by laboratory microscopists with additional reading on 10% of the samples by experienced supervisor. Additional study limitation have been added regarding Kato Katz technique in the discussion section.

When we talked about blinding in the discussion, it was about the cluster variation of STH infection prevalence that could not be explored before the end of the study, since the investigators should not know the clusters of high burden at baseline.

As part of Deworm3 design, a cross-sectional survey on 20,000 participants was done at baseline just after the LMC, with the aim of assessing the prevalence of STH with the PCR technique at baseline, after the 3 years of deworming intervention (to assess interruption of the transmission), and 2 years post intervention (to asses elimination). The baseline PCR results are also blinded until the end of intervention.

**Results**

-Does the analysis presented match the analysis plan?

-Are the results clearly and completely presented?

-Are the figures (Tables, Images) of sufficient quality for clarity?

Reviewer #4: The analysis presented match well the analysis plan. However, I have a concern regarding the age groups. Are there any rational for choosing these different age groups?

**Author response:** We thank the reviewer for this remark. In this study we needed to categorize individuals according to those exposed to the standard of care MDA against STH, which is school based. So we have the category of the pre-school aged children (PSAC=1-4 years old), the school-aged children (SAC=5-14 years old), then participants who are over 14 years old including adolescents and adults. Adolescents are considered in adults category since like adults they do not have access to standard school based MDA intervention.

The parenthesis in the tables should indicating the inclusion or exclusion of the numbers with parenthesis.

Why authors can't create more groups in adult group with aim to yield the ttrend association of hookworm infection with age if any? On Fig.4 it is not clear that hookworm infection increases with age.

**Author response:** We agree that Fig.4 does not show a clear increase of hookworm infection intensity with age. We do not have a linear and sharp increase, however for both sex, when we compare intensity of hookworm infection in children and in adults, we see that intensity is higher in adults. Looking at the statistics, we see on Table 3, a significantly increasing trend in intensity of hookworm infection between PSAC, SAC and adults (adults being the reference category). PSAC (1-4 years): Infection Intensity Ratio (IIR)= 0.1, 95%CI(0.0 - 0.3) p <0.001; SAC (5-14 years): IIR= 0.3 (0.1 – 0.7) p=0.01.

Is there any explanation related to the prevalence difference between male and female?

**Author response:** The prevalence difference is remarkable between 15-45 years old, with male more infected than females. That period corresponds to women reproductive age. The difference might be due to the routine deworming treatment received by women during antenatal care starting from the 2^nd^ trimester of pregnancy, a single dose of albendazole (400mg) or mebendazole (500mg), as recommended by WHO guidelines. At a population level that could lead to a significant difference in infection prevalence between male and female.

Reference: Preventive chemotherapy in human helminthiasis: coordinated use of anthelminthic drugs in control interventions ; a manual for health professionals and programme managers. Geneva: World Health Organization; 2006. 62 p

For Ascaris, here again there is a cluster increase of Ascaris around 10 years old, indicating no clear negative association between Ascaris infection with age. This is true only after 15 years old.

**Author response:** We thank the reviewer for this comment.

Which Hookworm species authors reported here?

**Author response:** We used Kato Katz technique in this study, and it could not allow to report differently hookworm species as eggs cannot be differentiated at microscopy.

**Conclusions**

-Are the conclusions supported by the data presented?

-Are the limitations of analysis clearly described?

-Do the authors discuss how these data can be helpful to advance our understanding of the topic under study?

-Is public health relevance addressed?

Reviewer #4: The conclusion supported the data presented partially. Indeed only hookworm infection was retained the attention of the authors. For limitation, author should elaborate on the limitation of Kato-katz as well as on available microscopic techniques including stool cultures which may change the current result particularly when hookworm is concern?

**Author response:** We thank the reviewer for this comment. Additional study limitation have been added regarding Kato Katz technique in the discussion section.

**Editorial and Data Presentation Modifications?**

Use this section for editorial suggestions as well as relatively minor modifications of existing data that would enhance clarity. If the only modifications needed are minor and/or editorial, you may wish to recommend “Minor Revision” or “Accept”.

Reviewer #4: Minor revision are needed,.the choice of age groups, the limitation of Kato-katz, and other stool microscopical testing.

**Summary and General Comments**

Use this section to provide overall comments, discuss strengths/weaknesses of the study, novelty, significance, general execution and scholarship. You may also include additional comments for the author, including concerns about dual publication, research ethics, or publication ethics. If requesting major revision, please articulate the new experiments that are needed.

Reviewer #4: This manuscript is well written and rThe author reports the already known factor associated with STH infection, but has an advantage with a high sample size. Minor chnages are required and can then been published in the PNTD. Indeed it provides a lot of update on the major STH infections.
